# Synthesis of indenones *via* photo-induced radical cascade cyclization of alkynes with alkyl halides

**DOI:** 10.1039/d5ra05436b

**Published:** 2025-09-17

**Authors:** Xiao Liu, Huadi Zhou, Xiaohu Yang, Zhen Wang

**Affiliations:** a The Fourth Clinical Medical School of Zhejiang Chinese Medical University Hangzhou Zhejiang China; b Zhejiang Hospital Hangzhou Zhejiang China; c Department of Pharmacy, Zhejiang Hospital Hangzhou Zhejiang China; d Hangzhou First People's Hospital Hangzhou Zhejiang China wangzhen@hospital.westlake.edu.cn

## Abstract

A radical cascade cyclization of aryl ynones with alkyl halides was established to synthesise alkylated indenones in yields of 43 to 83% under metal- and oxidant-free and room temperature conditions. The approach enables the construction of two C–C bonds through a XAT process using *n*Bu_3_N as XAT catalyst.

## Abstract 2

A radical cascade cyclization of aryl ynones with alkyl halides was established to synthesise alkylated indenones in yields of 43 to 83% under metal- and oxidant-free and room temperature conditions.

## Graphical abstract

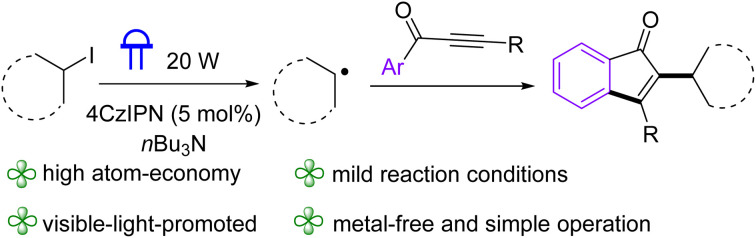

In organic synthesis, alkyl halides act as multifunctional precursors, able to form alkyl radicals and participate in numerous critical reactions.^1^ The halogen-atom transfer (XAT) method has become the most widely used strategy in recent years for generating alkyl radicals from alkyl halides.^2^ It involves the use of hydrogen abstraction reagents, often a radical intermediate, to abstract halogens from organic halides through homolytic C–X bond cleavage. Over the past decade in synthetic methodology, organic photochemistry has emerged as one of the most dynamic research areas.^3^ Spanning from ultraviolet to visible light, and transitioning from metal-based to organic photocatalysts, this field has seen a continuous surge of innovative reactions.^4^ In this context, Leonori and colleagues reported a novel strategy to activate organic halides using aminoalkyl radicals, generated by oxidation of simple amines, as halogen-atom transfer agents.^5^ Although this strategy has advanced the activation and conversion of alkyl halides to some extent since then, applying it to the production of high-value compounds remains urgent.^2,6^

Indenones and its derivatives play a significant role in many fields, including natural products, medicinal chemistry and pharmacology.^7^ For example, pauciflorol F and quadrangularin A, isolated from the stem bark of *Vatica pauciflora* and the stem of *Cissus quadrangularis*, respectively, are recognized as promising candidates for inhibiting cancer growth.^8^ The development of efficient synthetic methods for indenones continues to be a high-priority objective in organic chemistry research.^9^ Recently, the radical cascade cyclization of aryl ynones induced by radicals is one of the most effective approaches to access indenones.^10^ For instance, Pan, Yu and coworkers developed a cyclization of aryl ynones with alkanes to alkylated indenones, where the benzoyl radical generated from the radical initiators (BPO) abstracts hydrogen from the C(sp^3^)–H bond of alkanes to afford alkyl radicals (Scheme 1b).^10*c*^ Later, Yang and Yu established a photoinduced 4CzIPN-catalyzed cyclization of aryl ynones for the synthesis of indenones, using 4-alkyl-DHPs, prepared from aldehydes, as alkyl radical precursor (Scheme 1c).^10*d*^ However, these processes require stoichiometric radical initiators, high reaction temperature, and raw materials synthesized in multiple steps. Alkynes play a crucial role as building blocks in organic reactions and are commonly utilized in chemical manufacturing. Over the past few years, radical-initiated alkyne addition–cyclization processes have developed into a reliable method for the rapid formation of cyclic frameworks.^11^ However, visible-light promoted radical cyclization reaction of alkyl halides with aryl ynones for the synthesis of indenones is absent in the literature. Herein, we describe the photoinduced 4CzIPN-catalyzed radical annulation of ynones with alkyl halides to afford 2,3-difunctionalized indenone derivatives under metal-free and room temperature conditions (Scheme 1d).

**Scheme 1:**
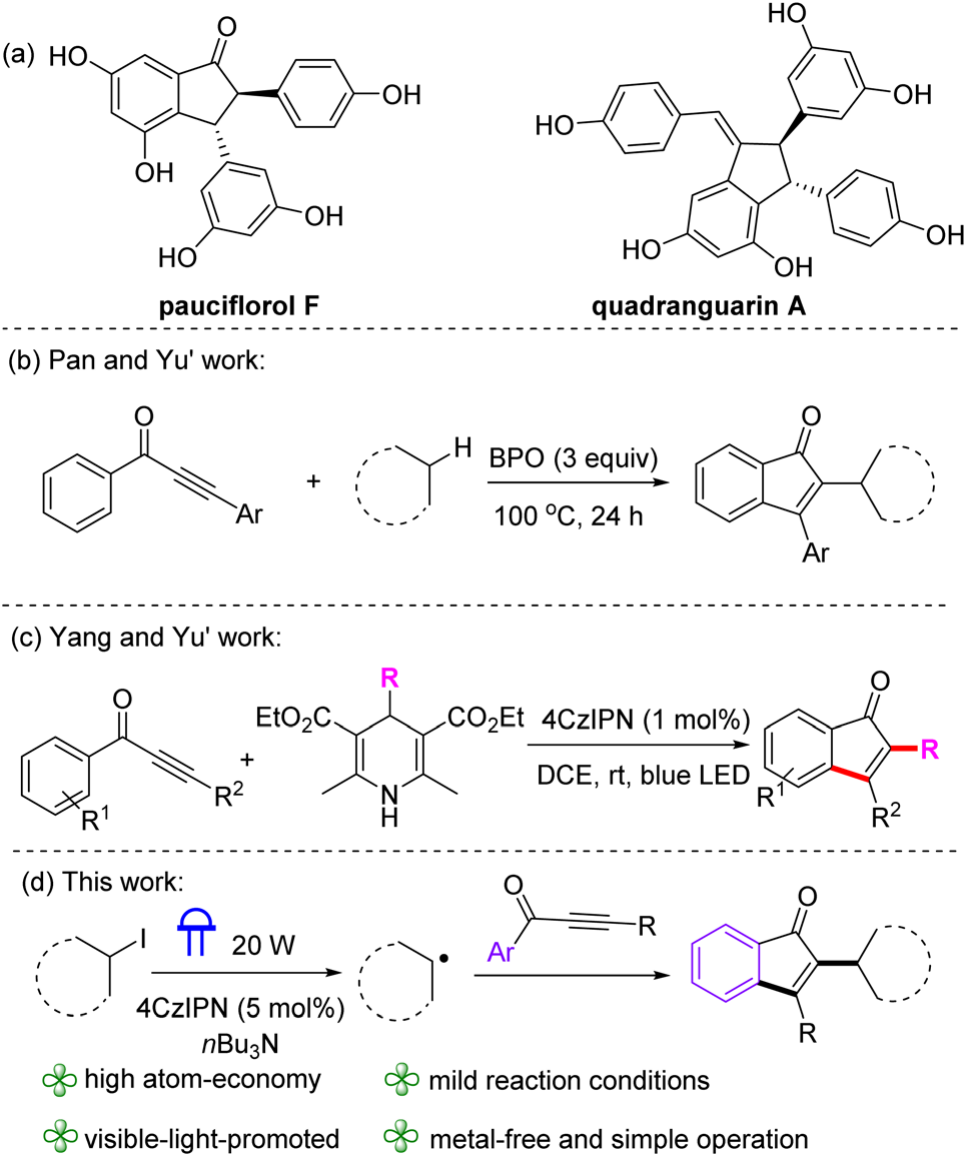
Background and this work.

To realize our initial idea, we selected model substrate 1a as radical acceptor and iodo-cyclohexan 2a as the precursors of alkyl radicals for the cascade. Inspired by the previous work, the 4CzIPN and *n*Bu_3_N were first chosen as photocatalyst and HAT-catalyst in EtOAc under blue light irradiation, generating 3a in 76% isolated yield (Table 1, entry 1). Employing Et_3_N, TMEDA, DIPEA and Na_2_CO_3_ resulted in reduced yields (Table 1, entries 2–5). We then turned our attention to studying the reaction in various solvents, including THF, DMSO, DMF, MeCN, and 1,4-dioxane (Table 1, entries 6–10). It is noteworthy that this reaction can not proceed in the absence of light or Bu_3_N (Table 1, entries 11 and 12). Meanwhile, cyclohexyl bromide instead of cyclohexyl iodine in this cascade cyclization afforded lower yield of 3a probably due to the higher homolytic BDE of C–Br bonds (entry 13).

**Table 1:** Optimization of the reaction conditions^a^

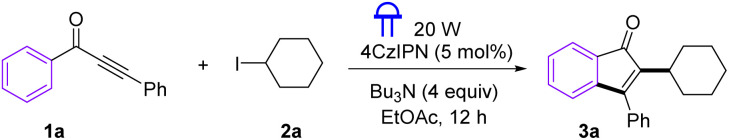			
Entry	Additive	Solvent	Yield^b^ (%)
1	Bu_3_N	EtOAc	76
2	Et_3_N	EtOAc	60
3	TMEDA	EtOAc	35
4	DIPEA	EtOAc	43
5	Na_2_CO_3_	EtOAc	nr
6	Bu_3_N	THF	63
7	Bu_3_N	DMSO	58
8	Bu_3_N	DMF	70
9	Bu_3_N	MeCN	55
10	Bu_3_N	1,4-Dioxane	57
11^b^	Bu_3_N	EtOAc	nr
12	—	EtOAc	nr
13^c^	Bu_3_N	EtOAc	52

a Reaction conditions: aryl ynones (1a, 0.20 mmol), 2a (0.5 mmol), 4CzIPN (5 mol%), *n*Bu_3_N (0.8 mmol) in EtOAc (2 mL) with the irradiation of 20 W blue LEDs at room temperature for 12 h. Yields are given for isolated products, nr = no reaction.

b Reaction was conducted in the absence of a light source.

c Cyclohexyl bromide instead of cyclohexyl iodine.

With the optimized conditions in hand, the scope of aryl ynones and cyclohexane to synthesize was first explored (Scheme 2). The aryl ynones with a chlorine atom on the *para*-position of Ar ring was well suitable for this cascade, affording the expected product 3b in 63% yield. Next, we examined the effect of introducing electron-donating groups at the *para*-position of the Ar ring on the conversion. For example, the substrates featuring Me, OMe, and Ph groups were all compatible in the cascade reactions, generating the corresponding indenone derivatives in 50–71% yields (3c: 66%; 3e: 71%). Delightfully, the 3-substituted aryl ynones (1f and 1g) afforded products 3f and 3g in 43% and 64% yields, respectively. When a series of aryl ynones with substitution on the Ar1 ring were tested. The reaction tolerated electron-rich *para*-substituted groups, successfully affording the target compounds 3h–3m in 55–75% yields. However, electron-poor group failed to obtain target product 3n, which may be attributed to the fact that the intermediate of vinyl radicals tends to react more with electron rich aromatic rings. *Ortho*-fluorination and methylation of aryl ynones (1o and 1p) obtained alkylated indenones 3o and 3p in 79% and 73% yields. Interestingly, only a single alkylated product 3q was isolated when *meta*-Cl substituted Ar1 ring was applied as substrate. The capacity of the present cascade alkylated cyclization reaction was also illustrated by the scope of aryl ynones with other organic halide coupling partners. Iodo-cyclopentan was compatible with this procedure, providing the desired products 3r in 83 yield%. In addition, other organic halides such as 2-iodobutane and 2-iodopropane also proceeded smoothly to produce the products in good yields (3s: 46%; 3t: 72%).

**Scheme 2:**
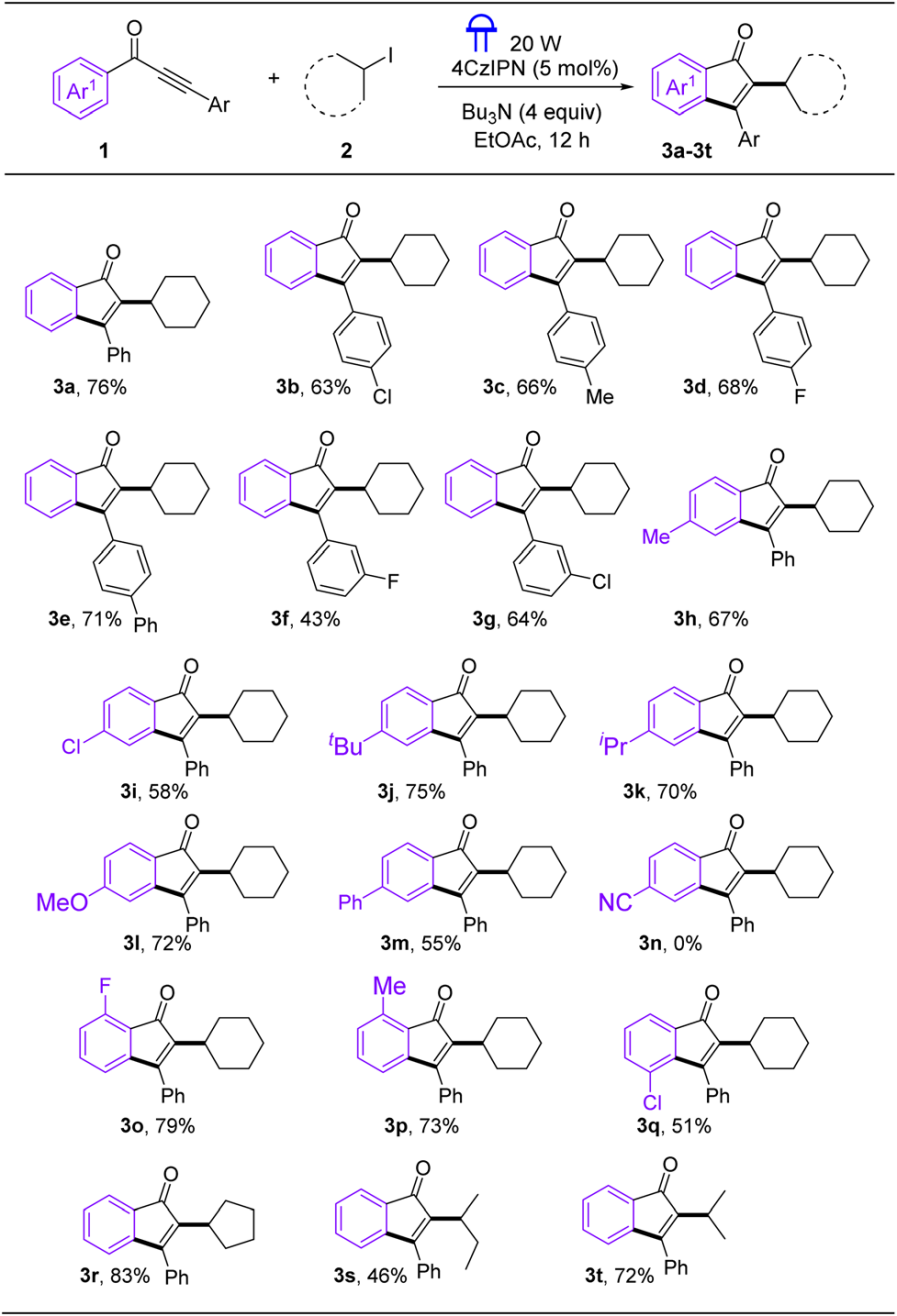
Substrate scope.^*a a*^Reaction conditions: aryl ynones 1 (0.20 mmol), 2 (0.5 mmol), 4CzIPN (5 mol%) and *n*Bu_3_N (0.8 mmol) in EtOAc (2 mL) with the irradiation of 20 W blue LEDs at room temperature for 12 h.

Mechanistic experiments were performed to gain deeper insights into the reaction pathways. When aryl ynones reacted with cyclohexane under the optimized reaction conditions with the addition of 2 equivalents of 2,2,6,6-tetramethyl-1-oxylpiperidine (TEMPO), a well known radical scavenger, no alkylated indenone 3a was obtained. Meanwhile, the traped product A, generated from the coupling between TEMPO and the cyclohexyl radical, was identified by HR-MS spectrometry (Scheme 3a). These results strongly support a radical-mediated pathway for the cascade reaction. Following this, light modulation experiments (on/off cycling) were conducted to evaluate light irradiation effects. The reaction occurred efficiently only under light irradiation; its absence suppressed the process, indicating that light is essential (Scheme 3b). The desired product 3a was also obtained when the reaction was conducted using either K_2_S_2_O_8_ or Na_2_S_2_O_8_ as an oxidant in the absence of light, and this reslut indicates that the involvement of the α-amino alkyl radical under our reaction conditions (Scheme 3c).^12^

**Scheme 3:**
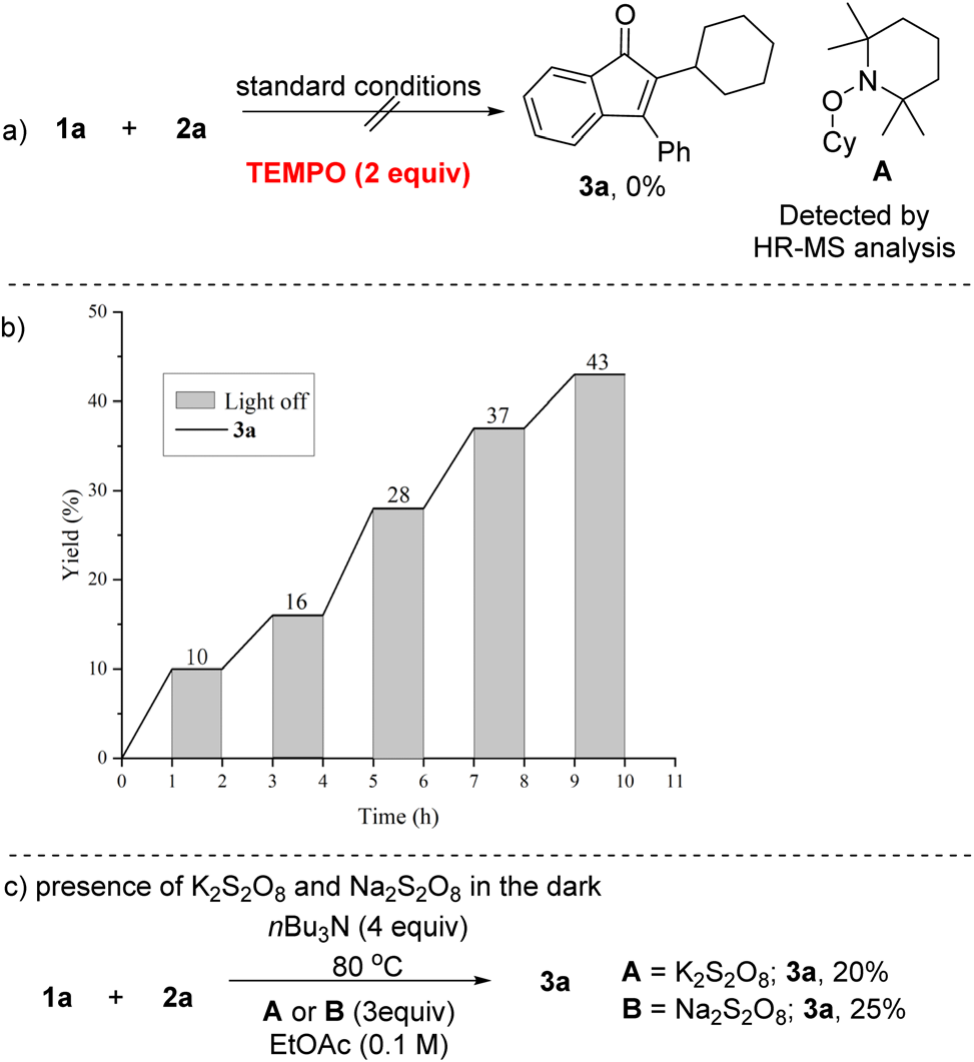
Control experiments.

Based on the aforementioned experimental results and related literature reports,^6,12^ a plausible radical mechanism for the formation of indenone was proposed (Scheme 4). Irradiation of the 4CzIPN with blue LEDs generates the excited state 4CzIPN*, and a single electron transfer (SET) with *n*Bu_3_N leads to radical intermediate i. Then, intermediate i abstracts iodine atom from 2a*via* XAT process, affording alkyl radical intermediate ii. Subsequently, intermediate ii undergoes free radical addition and intramolecular cyclization to obtain another intermediate iii, followed by XAT/SET process between intermediate iii and another molecule of iodoalkane to afford the cationic intermediate iv. Finally, the desired product 3a is provided by deprotonation of the intermediate iv.

**Scheme 4:**
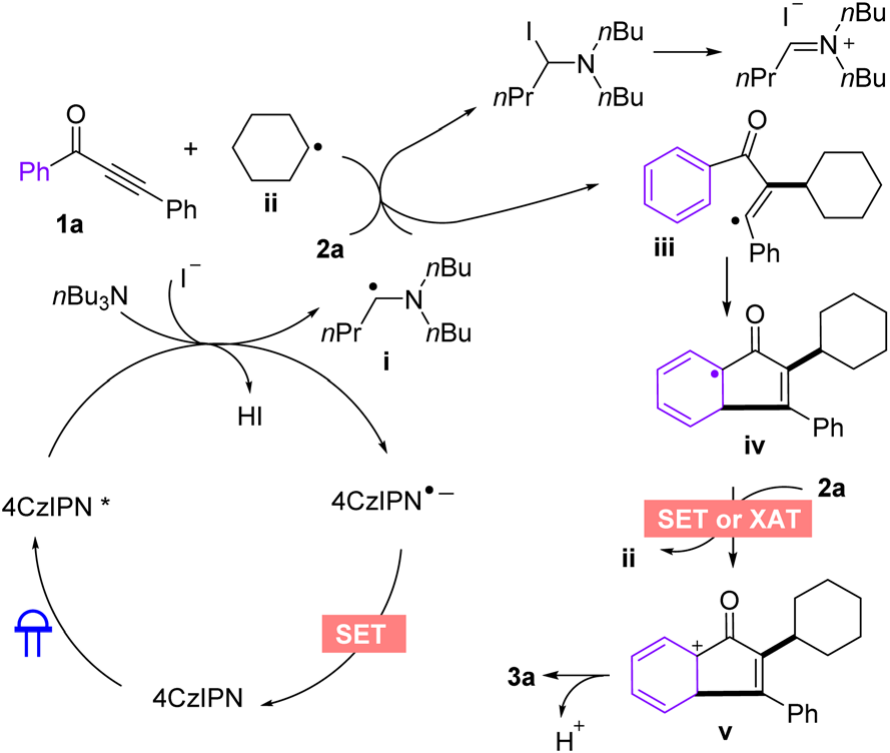
Proposed mechanism.

In conclusion, we have achieved a new reaction that allows the synthesis of alkylated indenone derivatives *via* a 4CzIPN-catalyzed XAT, alkyl radical addition, annulation pathway. The approach enables the construction of two C–C bonds under metal- and external oxidant-free conditions through a XAT process using *n*Bu_3_N as XAT catalyst. Abundant, stable, and cost-effective organic halides such as iodo-cyclohexan and iodo-cyclopentan are onboard at ambient conditions. In addition, the current photo-induced XAT-catalyzed alkylative cyclization exhibits broad substrate scope (–F, –Cl, –Me, –OMe, –Ph, ^i^Pr) and versatility. The investigation of further applications for this transformation is ongoing in our laboratory.

## Conflicts of interest

There are no conflicts to declare.

## Supplementary Material

RA-015-D5RA05436B-s001

## Data Availability

The data underlying this study are available in the published article and its SI. See DOI: https://doi.org/10.1039/d5ra05436b.
