# Supplementary material for: Synthesis of indenones via photo-induced radical cascade cyclization of alkynes with alkyl halides
Source: RSC Adv. 2025 Sep 17;15(41):33962–5. doi: 10.1039/d5ra05436b (PMC12441893; doi:10.1039/d5ra05436b)

Supplementary Information (SI) for RSC Advances.
This journal is © The Royal Society of Chemistry 2025

Supporting Information for

Synthesis of indenones via photo-induced radical cascade cyclization of alkynes with alkyl halides

Xiao Liu,[a,b†] Huadi Zhou,[a,b†] Xiaohu Yang,[b†] Zhen Wang,*[c]

[a] *The Fourth Clinical Medical School of Zhejiang Chinese Medical University, Hangzhou, China.*
[b] *Department of Pharmacy, Zhejiang Hospital, Hangzhou, Zhejiang 310013, P. R. China.*
[c] *Hangzhou First People's Hospital, Hangzhou, China.*

Table of Contents for Supporting Information

1. General considerations……………………………………………………………S2
2. General procedure for the synthesis of **3a**……………………………………S2
3. Radical trapping experiment…………………………………………………………S3
4. Characterization data of products..S4
5. NMR spectra of the products..S13

1. General considerations

All reactions were carried out under air. ^{1}H NMR and ^{13}C NMR spectra were measured on a Bruker Avance NMR spectrometer (600 MHz/150 MHz/565 NMR) in $CDCl_3$ as solvent and recorded in ppm relative to internal tetramethylsilane standard. ^{1}H NMR data are reported as follows: δ, chemical shift; coupling constants (J are given in Hertz, Hz) and integration. Abbreviations to denote the multiplicity of a particular signal were s (singlet), d (doublet), t (triplet), q (quartet), dd (doublet of doublets) and m (multiplet). All reactions were performed under nitrogen. The 20 W blue LED was purchased directly from the Taobao, Alibaba.

2. General procedure for the synthesis of 3a

To an oven-dried 20 mL Schlenk tube charged with aryl ynones **1** (0.2 mmol), alkyl halides (**2**, 0.5 mmol), tributylamine (0.8 mmol) and 4CzIPN (5 mol%). The tube was degassed by alternating vacuum evacuation (3 min) and nitrogen backfill for three times. Then, EtOAc (2 mL) was injected into the tube. The resulting mixture was stirred at room temperature with the irradiation of 20 W blue LED for 12 hours. After completion of reaction as monitored by TLC analysis, the mixture was cooled to room temperature, and the solvent was then removed under vacuo. After the solvent was removed under reduced pressure, the crude product was

further purified by flash chromatography (silica gel, petroleum ether/ethyl acetate) to give the corresponding product **3a**.

3. Radical trapping experiment

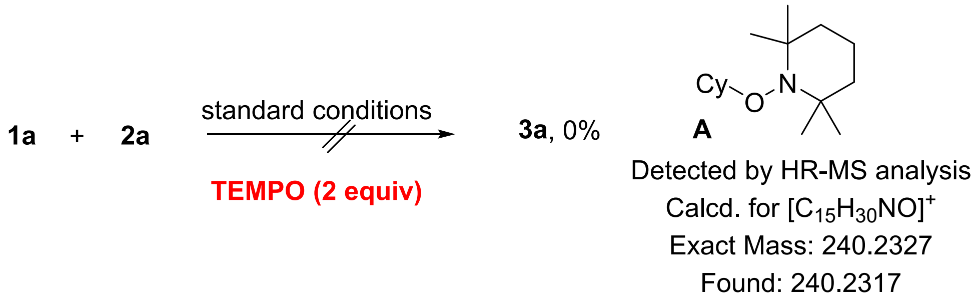

TEMPO (2 equiv) was subjected to the standard conditions and reacted for 12 hours. The reaction was fully inhibited and no desired product could be detected. The TEMPO-adduct could be detected by HRMS analysis of the reaction system (see below).

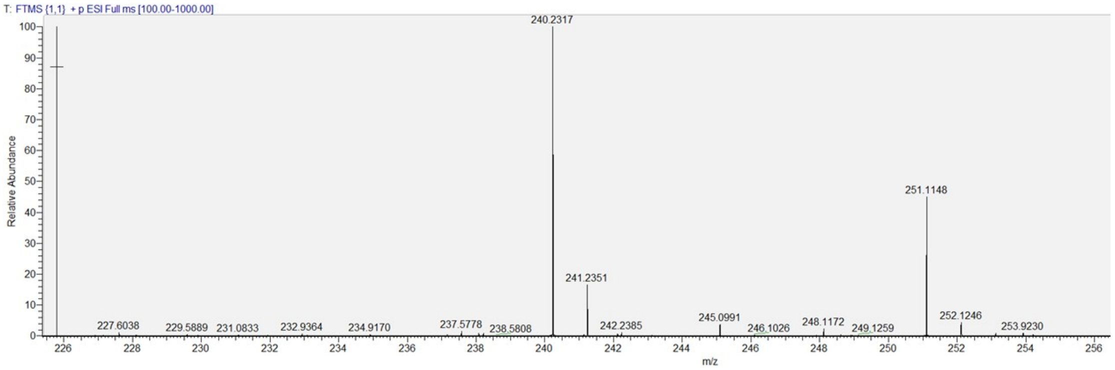

4. Characterization data of products

2-cyclohexyl-3-phenyl-1*H*-inden-1-one (3a)[1]

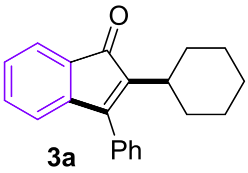

The product purified by flash column chromatography on silica gel (PE/EA = 50/1) to afford the **3a** as a yellow oil (76% yield). ^{1}H NMR (600 MHz, $CDCl_3$) δ 7.51 (t, *J* = 7.4 Hz, 2H), 7.46 (d, *J* = 7.4 Hz, 1H), 7.45 – 7.41 (m, 1H), 7.40 – 7.36 (m, 2H), 7.28 – 7.25 (m, 2H), 7.18 (t, *J* = 7.3 Hz, 1H), 6.89 (d, *J* = 7.2 Hz, 1H), 2.53 – 2.44 (m, 1H), 1.85 (ddd, *J* = 25.1, 12.6, 3.3 Hz, 2H), 1.74 (d, *J* = 13.1 Hz, 2H), 1.65-1.59 (m, 2H), 1.30 – 1.22 (m, 2H), 1.19 (ddd, *J* = 13.1, 8.2, 3.3 Hz, 2H). ^{13}C NMR (150 MHz, $CDCl_3$) δ 198.21, 154.66, 145.81, 139.05, 133.12, 130.94, 128.90, 128.72, 128.19, 127.94, 122.19, 120.45, 35.94, 31.11, 26.55, 25.76.

3-(4-Chlorophenyl)-2-cyclohexyl-1*H*-inden-1-one (3b)[1]

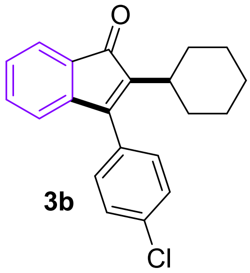

The product purified by flash column chromatography on silica gel (PE/EA = 50/1) to afford the **3b** as a yellow oil (63% yield). ^{1}H NMR (600 MHz, $CDCl_3$) δ 7.42 (dd, *J* = 8.7, 2.0 Hz, 2H), 7.36 (d, *J* = 7.0 Hz, 1H), 7.29 – 7.23 (m, 2H), 7.20 (dd, *J* = 11.0, 4.2 Hz, 1H), 7.12 (t, *J* = 7.2 Hz, 1H), 6.77 (d, *J* = 7.2 Hz, 1H), 2.35 (tt, *J* = 12.1, 3.4 Hz, 1H), 1.75 (ddd, *J* = 25.1, 12.6, 3.2 Hz, 2H), 1.67 (d, *J* = 13.1 Hz, 2H), 1.57 (d, *J* = 12.5 Hz, 1H), 1.51 – 1.46 (m, 2H), 1.18 (dd, *J* = 14.0, 11.3 Hz, 1H), 1.14 – 1.05 (m, 2H). ^{13}C NMR (150 MHz, $CDCl_3$) δ 197.83 (s), 153.35 (s), 145.46 (s), 139.46 (s), 134.85 (s), 133.24 (s), 131.53 (s), 130.75 (s), 129.36 (s), 129.09 (s), 128.38 (s), 122.39 (s), 120.25 (s), 77.25 (s), 77.04 (s), 76.83 (s), 36.02 (s), 31.11 (s), 26.53 (s), 25.72 (s).

2-Cyclohexyl-3-(*p*-tolyl)-1*H*-inden-1-one (3c)[1]

The product purified by flash column chromatography on silica gel (PE/EA = 50/1) to afford the **3c** as a yellow oil (66% yield). ^{1}H NMR (600 MHz, $CDCl_3$) δ 7.34 (d, *J* = 7.0 Hz, 1H), 7.25 (d, *J* = 8.0 Hz, 2H), 7.22 –

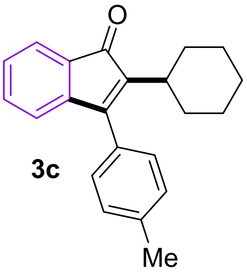

7.16 (m, 3H), 7.10 (t, *J* = 7.3 Hz, 1H), 6.83 (d, *J* = 7.2 Hz, 1H), 2.41 (ddd, *J* = 12.1, 8.7, 3.5 Hz, 1H), 2.38 (s, 3H), 1.79 (qd, *J* = 12.7, 3.2 Hz, 2H), 1.67 (d, *J* = 13.1 Hz, 2H), 1.57 (d, *J* = 12.6 Hz, 1H), 1.51 - 1.49 (m, 1H), 1.22 – 1.16 (m, 2H), 1.15 – 1.07 (m, 2H). ^{13}C NMR (151 MHz, $CDCl_3$) δ 198.29 (s), 154.79 (s), 145.80 (s), 138.97 (s), 138.75 (s), 133.04 (s), 131.08 (s), 130.10 (s), 129.41 (s), 128.12 (s), 127.89 (s), 122.08 (s), 120.46 (s), 35.97 (s), 31.08 (s), 26.58 (s), 25.78 (s), 21.48 (s).

2-Cyclohexyl-3-(4-fluorophenyl)-1*H*-inden-1-one (3d)[1]

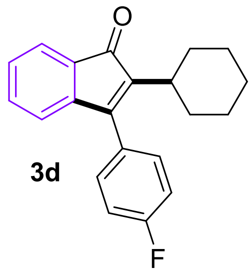

3d

The product purified by flash column chromatography on silica gel (PE/EA = 50/1) to afford the **3d** as a yellow oil (68% yield). ^{1}H NMR (600 MHz, $CDCl_3$) δ 7.35 (d, *J* = 7.0 Hz, 1H), 7.32 – 7.23 (m, 2H), 7.22 – 7.17 (m, 1H), 7.17 – 7.01 (m, 3H), 6.78 (d, *J* = 7.2 Hz, 1H), 2.36 (tt, *J* = 12.1, 3.4 Hz, 1H), 1.76 (ddd, *J* = 25.0, 12.6, 3.2 Hz, 2H), 1.67 (d, *J* = 13.1 Hz, 2H), 1.57 (d, *J* = 9.4 Hz, 1H), 1.50 – 1.47 (m, 1H), 1.23 – 1.14 (m, 2H), 1.10 (ddt, *J* = 16.3, 6.9, 3.2 Hz, 2H). ^{13}C NMR (151 MHz, $CDCl_3$) δ 197.94 (s), 162.93 (d, *J* = 247.5 Hz), 153.62 (s), 145.62 (s), 139.24 (s), 133.19 (s), 130.82 (s), 129.85 (d, *J* = 7.8 Hz), 129.03 (d, *J* = 3.4 Hz), 128.32 (s), 122.29 (s), 120.27 (s), 115.93 (d, *J* = 21 Hz), 36.01 (s), 31.10 (s), 26.55 (s), 25.74 (s).

3-([1,1'-Biphenyl]-4-yl)-2-cyclohexyl-1*H*-inden-1-one (3e)[1]

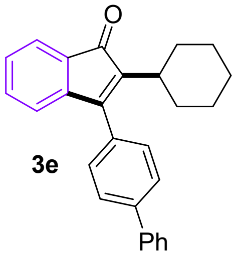

3e

The product purified by flash column chromatography on silica gel (PE/EA = 50/1) to afford the **3e** as a yellow oil (71% yield). ^{1}H NMR (600 MHz, $CDCl_3$) δ 7.81 – 7.64 (m, 2H), 7.60 (dd, *J* = 8.2, 1.1 Hz, 2H), 7.40 (ddd, *J* = 21.7, 10.5, 4.3 Hz, 5H), 7.32 (t, *J* = 7.4

Hz, 1H), 7.21 (td, *J* = 7.6, 1.1 Hz, 1H), 7.15 – 7.07 (m, 1H), 6.89 (d, *J* = 7.2 Hz, 1H), 2.54 – 2.40 (m, 1H), 1.82 (qd, *J* = 12.7, 3.2 Hz, 2H), 1.69 (d, *J* = 13.1 Hz, 2H), 1.58 (d, *J* = 12.5 Hz, 1H), 1.55 – 1.52 (m, 2H), 1.23 – 1.19 (m, 1H), 1.17 – 1.11 (m, 2H). ^{13}C NMR (150 MHz, $CDCl_3$) δ 197.10 (s), 153.26 (s), 144.62 (s), 140.72 (s), 139.35 (s), 138.10 (s), 132.09 (s), 130.95 (s), 129.98 (s), 127.91 (s), 127.44 (s), 127.19 (s), 126.71 (s), 126.33 (s), 126.06 (s), 121.19 (s), 119.46 (s), 35.01 (s), 30.08 (s), 25.53 (s), 24.73 (s).

2-Cyclohexyl-3-(3-fluorophenyl)-1*H*-inden-1-one (3f)[1]

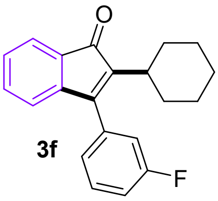

The product purified by flash column chromatography on silica gel (PE/EA = 50/1) to afford the **3f** as a yellow oil (43% yield). ^{1}H NMR (600 MHz, $CDCl_3$) δ 7.54 – 7.46 (m, 1H), 7.44 (d, *J* = 7.0 Hz, 1H), 7.29 – 7.24 (m, 1H), 7.25 – 7.11 (m, 3H), 7.08 (d, *J* = 9.2 Hz, 1H), 6.86 (d, *J* = 7.2 Hz, 1H), 2.45 (tt, *J* = 12.1, 3.3 Hz, 1H), 1.83 (ddd, *J* = 25.0, 12.6, 3.0 Hz, 2H), 1.75 (d, *J* = 13.0 Hz, 2H), 1.65 (d, *J* = 12.3 Hz, 1H), 1.57 (d, *J* = 11.9 Hz, 1H), 1.29 – 1.15 (m, 4H). ^{13}C NMR (150 MHz, $CDCl_3$) δ 197.83 (s), 162.90 (d, *J* = 247.3 Hz), 153.15 (s), 145.45 (s), 139.58 (s), 135.26 (d, *J* = 7.7 Hz), 133.30 (s), 130.66 (s), 130.48 (d, *J* = 8.0 Hz), 128.38 (s), 123.76 (d, *J* = 2.7 Hz), 122.43 (s), 120.31 (s), 115.85 (d, *J* = 20.9 Hz), 114.98 (d, *J* = 21.9 Hz), 35.99 (s), 31.09 (s), 26.51 (s), 25.72 (s).

3-(3-Chlorophenyl)-2-cyclohexyl-1*H*-inden-1-one (3g)[1]

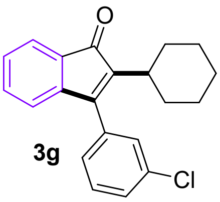

The product purified by flash column chromatography on silica gel (PE/EA = 50/1) to afford the **3g** as a yellow oil (64% yield). ^{1}H NMR (600 MHz, $CDCl_3$) δ 7.53 – 7.41 (m, 3H), 7.37 (d, *J* = 1.6 Hz, 1H), 7.30 – 7.23 (m, 2H), 7.20 (dd, *J* = 10.9, 3.8 Hz, 1H), 6.85 (d, *J* = 7.2 Hz, 1H), 2.43 (tt, *J* = 12.1, 3.4 Hz, 1H), 1.82 (ddd, *J* = 24.9, 12.5, 3.1 Hz,

2H), 1.75 (d, *J* = 13.1 Hz, 2H), 1.65 (d, *J* = 12.4 Hz, 1H), 1.60 – 1.53 (m, 2H), 1.26 (dd, *J* = 9.4, 3.0 Hz, 1H), 1.23 – 1.17 (m, 2H). ^{13}C NMR (150 MHz, $CDCl_3$) δ 197.77 (s), 152.99 (s), 145.42 (s), 139.71 (s), 134.96 (s), 134.76 (s), 133.32 (s), 130.63 (s), 130.14 (s), 129.01 (s), 128.41 (s), 127.90 (s), 126.20 (s), 122.46 (s), 120.29 (s), 36.02 (s), 31.12 (s), 26.50 (s), 25.72 (s).

2-Cyclohexyl-5-methyl-3-phenyl-1*H*-inden-1-one (3h)[1]

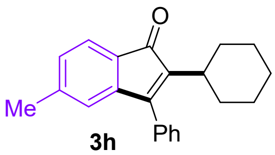

3h

The product purified by flash column chromatography on silica gel (PE/EA = 50/1) to afford the **3h** as a yellow oil (67% yield). ^{1}H NMR (600 MHz, $CDCl_3$) δ 7.52 (t, *J* = 7.4 Hz, 2H), 7.47 (dd, *J* = 4.9, 3.7 Hz, 1H), 7.37 (dd, *J* = 5.1, 3.2 Hz, 2H), 7.32 (d, *J* = 7.2 Hz, 1H), 6.96 (d, *J* = 7.2 Hz, 1H), 6.68 (s, 1H), 2.45 (tt, *J* = 12.1, 3.4 Hz, 1H), 2.28 (s, 3H), 1.83 (qd, *J* = 12.6, 3.2 Hz, 2H), 1.76 – 1.70 (m, 2H), 1.63 (d, *J* = 12.5 Hz, 1H), 1.56 (s, 2H), 1.26 – 1.23 (m, 1H), 1.21 – 1.13 (m, 2H). ^{13}C NMR (150 MHz, $CDCl_3$) δ 197.94 (s), 154.18 (s), 146.29 (s), 144.03 (s), 139.49 (s), 133.26 (s), 128.78, 128.70, 128.55 (s), 128.10, 127.99, 122.29 (s), 121.76 (s), 35.98 (s), 31.13 (s), 26.56 (s), 25.77 (s), 22.00 (s).

5-Chloro-2-cyclohexyl-3-phenyl-1*H*-inden-1-one (3i)[1]

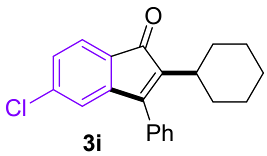

3i

The product purified by flash column chromatography on silica gel (PE/EA = 50/1) to afford the **3i** as a yellow oil (58% yield). ^{1}H NMR (600 MHz, $CDCl_3$) δ 7.53 (t, *J* = 7.4 Hz, 2H), 7.48 (d, *J* = 7.3 Hz, 1H), 7.36 (d, *J* = 7.5 Hz, 3H), 7.16 (dd, *J* = 7.6, 1.5 Hz, 1H), 6.86 (d, *J* = 1.5 Hz, 1H), 2.48 (tt, *J* = 12.1, 3.4 Hz, 1H), 1.83 (ddd, *J* = 25.0, 12.6, 3.1 Hz, 2H), 1.74 (d, *J* = 13.1 Hz, 2H), 1.64 (d, *J* = 12.4 Hz, 1H), 1.28 – 1.25 (m, 1H), 1.29 – 1.14 (m, 4H). ^{13}C NMR (150 MHz, $CDCl_3$) δ 196.65 (s), 153.53 (s), 147.79 (s), 140.39 (s), 139.36 (s), 132.49 (s), 129.19 (s), 129.07 (s), 128.91 (s), 127.85 (s), 127.74 (s), 123.12 (s), 121.24 (s), 36.05 (s), 31.03 (s), 26.48 (s), 25.71 (s).

5-(*tert*-Butyl)-2-cyclohexyl-3-phenyl-1*H*-inden-1-one (3j)[1]

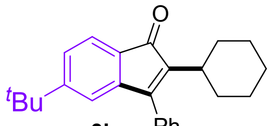

3j

The product purified by flash column chromatography on silica gel (PE/EA = 50/1) to afford the **3j** as a yellow oil (75% yield). ^{1}H NMR (600 MHz, $CDCl_3$) δ 7.53 (t, *J* = 7.4 Hz, 2H), 7.47 (t, *J* = 7.4 Hz, 1H), 7.43 – 7.34 (m, 3H), 7.18 (dd, *J* = 7.5, 1.5 Hz, 1H), 6.91 (d, *J* = 1.1 Hz, 1H), 2.45 (tt, *J* = 12.1, 3.4 Hz, 1H), 1.85 (qd, *J* = 12.6, 3.2 Hz, 2H), 1.73 (d, *J* = 13.1 Hz, 2H), 1.63 (d, *J* = 12.0 Hz, 1H), 1.29 – 1.25 (m, 11H), 1.18 (dd, *J* = 14.5, 11.2 Hz, 2H). ^{13}C NMR (150 MHz, $CDCl_3$) δ 197.92 (s), 157.31 (s), 154.42 (s), 145.89 (s), 139.54 (s), 133.24 (s), 128.79, 128.72, 127.99 (s), 124.39 (s), 122.10 (s), 118.23 (s), 36.00 (s), 35.40 (s), 31.08 (s), 26.57 (s), 25.77 (s).

2-Cyclohexyl-5-isopropyl-3-phenyl-1*H*-inden-1-one (3k)[1]

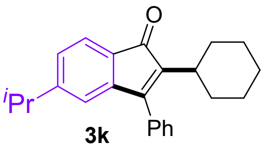

3k

The product purified by flash column chromatography on silica gel (PE/EA = 50/1) to afford the **3k** as a yellow oil (70% yield). ^{1}H NMR (600 MHz, $CDCl_3$) δ 7.53 (dd, *J* = 10.2, 4.6 Hz, 2H), 7.50 – 7.44 (m, 1H), 7.40 – 7.32 (m, 3H), 7.02 (dd, *J* = 7.4, 1.0 Hz, 1H), 6.73 (d, *J* = 1.2 Hz, 1H), 2.83 (dt, *J* = 13.8, 6.9 Hz, 1H), 2.45 (tt, *J* = 12.1, 3.4 Hz, 1H), 1.84 (qd, *J* = 12.7, 3.2 Hz, 2H), 1.73 (d, *J* = 13.1 Hz, 2H), 1.63 (d, *J* = 12.6 Hz, 1H), 1.56 (d, *J* = 12.6 Hz, 1H), 1.29 – 1.22 (m, 3H), 1.19 (s, 3H), 1.18 (s, 3H), 1.16 (t, *J* = 3.9 Hz, 1H). ^{13}C NMR (150 MHz, $CDCl_3$) δ 197.93 (s), 155.15 (s), 154.26 (s), 146.26 (s), 139.53 (s), 133.27 (s), 128.99 (s), 128.78, 128.71, 128.00 (s), 125.50 (s), 122.43 (s), 119.38 (s), 35.99 (s), 34.69 (s), 31.11 (s), 26.57 (s), 25.78 (s), 23.68 (s).

2-cyclohexyl-5-methoxy-3-phenyl-1*H*-inden-1-one (3l)[1]

The product purified by flash column chromatography on silica gel (PE/EA = 50/1) to afford the **3l** as a yellow oil (72% yield).

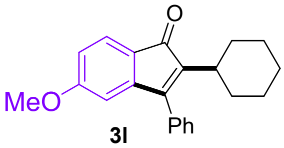

3l

^{1}H NMR (600 MHz, $CDCl_3$) δ 7.42 (t, *J* = 7.4 Hz, 2H), 7.37 (t, *J* = 7.4 Hz, 1H), 7.32 (d, *J* = 7.9 Hz, 1H), 7.30 – 7.26 (m, 2H), 6.49 (dd, *J* = 8.0, 2.1 Hz, 1H), 6.37 (d, *J* = 2.1 Hz, 1H), 3.71 (s, 3H), 2.39 (tt, *J* = 12.1, 3.4 Hz, 1H), 1.76 (qd, *J* = 12.7, 3.1 Hz, 2H), 1.66 (d, *J* = 13.0 Hz, 2H), 1.56 (d, *J* = 9.2 Hz, 1H), 1.50 (d, *J* = 11.9 Hz, 2H), 1.18 (dt, *J* = 11.6, 3.1 Hz, 1H), 1.14 – 1.06 (m, 2H). ^{13}C NMR (150 MHz, $CDCl_3$) δ 196.81 (s), 164.27 (s), 152.42 (s), 148.54 (s), 140.68 (s), 133.06 (s), 128.77 (s), 128.70 (s), 128.05 (s), 124.06 (s), 123.74 (s), 109.73 (s), 109.45 (s), 55.67 (s), 36.10 (s), 31.10 (s), 26.55 (s), 25.77 (s).

2-Cyclohexyl-3,5-diphenyl-1*H*-inden-1-one (3m)[1]

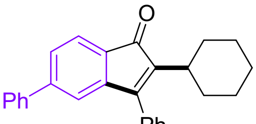

3m

The product purified by flash column chromatography on silica gel (PE/EA = 70/1) to afford the **3m** as a yellow oil (55% yield). ^{1}H NMR (600 MHz, $CDCl_3$) δ 7.51 – 7.41 (m, 5H), 7.39 (dd, *J* = 10.5, 4.3 Hz, 1H), 7.36 – 7.29 (m, 5H), 7.27 (dd, *J* = 8.3, 6.4 Hz, 1H), 7.01 (d, *J* = 1.1 Hz, 1H), 2.42 (tt, *J* = 12.1, 3.4 Hz, 1H), 1.79 (ddd, *J* = 25.1, 12.6, 3.2 Hz, 2H), 1.69 – 1.65 (m, 2H), 1.57 (d, *J* = 12.4 Hz, 2H), 1.51 (s, 1H), 1.20 – 1.16 (m, 1H), 1.15 – 1.06 (m, 2H). ^{13}C NMR (150 MHz, $CDCl_3$) δ 197.79 (s), 154.16 (s), 146.68 (s), 146.47 (s), 140.55 (s), 139.91 (s), 133.08 (s), 129.75 (s), 128.94, 128.82, 128.81, 128.10, 128.01, 127.16, 126.80 (s), 122.62 (s), 119.76 (s), 36.06 (s), 31.12 (s), 26.57 (s), 25.78 (s).

2-Cyclohexyl-7-fluoro-3-phenyl-1*H*-inden-1-one (3o)[1]

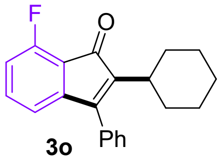

3o

The product purified by flash column chromatography on silica gel (PE/EA = 50/1) to afford the **3o** as a yellow oil (79% yield). ^{1}H NMR (600 MHz, $CDCl_3$) δ 7.44 (t, *J* = 7.3 Hz, 2H), 7.39 (t, *J* = 7.4 Hz, 1H), 7.30 – 7.26 (m, 2H), 7.18 (ddd, *J* = 12.2, 6.6, 3.8 Hz, 1H), 6.77 (t, *J*

= 8.5 Hz, 1H), 6.60 (d, *J* = 7.2 Hz, 1H), 2.38 (tt, *J* = 12.1, 3.4 Hz, 1H), 1.79 – 1.73 (m, 2H), 1.66 (d, *J* = 13.0 Hz, 2H), 1.56 (d, *J* = 12.3 Hz, 2H), 1.49 (s, 1H), 1.10 (ddd, *J* = 22.5, 15.3, 8.9 Hz, 3H). ^{13}C NMR (151 MHz, $CDCl_3$) δ 194.28 (s), 157.55 (d, *J* = 261 Hz), 153.95 (s), 148.05 (s), 139.71 (s), 135.51 (d, *J* = 8.4 Hz), 132.80 (s), 129.03 (s), 128.77 (s), 127.98 (s), 117.6 (d, *J* = 21 Hz), 116.91 (s), 35.99 (s), 31.00 (s), 26.52 (s), 25.73 (s).

2-Cyclohexyl-7-methyl-3-phenyl-1*H*-inden-1-one (3p)[1]

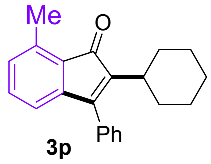

3p

The product purified by flash column chromatography on silica gel (PE/EA = 50/1) to afford the **3p** as a yellow oil (73% yield). ^{1}H NMR (600 MHz, $CDCl_3$) δ 7.50 (t, *J* = 7.4 Hz, 2H), 7.47 – 7.42 (m, 1H), 7.38 – 7.33 (m, 2H), 7.12 (t, *J* = 7.5 Hz, 1H), 6.94 (d, *J* = 7.8 Hz, 1H), 6.70 (d, *J* = 7.2 Hz, 1H), 2.54 (s, 3H), 2.45 (tt, *J* = 12.1, 3.5 Hz, 1H), 1.84 (qd, *J* = 12.6, 3.2 Hz, 2H), 1.73 (d, *J* = 13.0 Hz, 2H), 1.63 (d, *J* = 12.3 Hz, 1H), 1.58 – 1.56 (m, 2H), 1.24 – 1.13 (m, 3H). ^{13}C NMR (150 MHz, $CDCl_3$) δ 199.59 (s), 153.63 (s), 146.28 (s), 138.91 (s), 137.22 (s), 133.31 (s), 132.34 (s), 131.51 (s), 128.69, 128.62, 128.07 (s), 127.16 (s), 118.42 (s), 35.93 (s), 31.18 (s), 26.60 (s), 25.85 (s), 17.14 (s).

4-Chloro-2-cyclohexyl-3-phenyl-1*H*-inden-1-one (3q)[1]

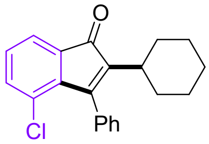

3q

The product purified by flash column chromatography on silica gel (PE/EA = 50/1) to afford the **3q** as a yellow solid (51% yield). ^{1}H NMR (600 MHz, $CDCl_3$) δ 7.48 – 7.40 (m, 3H), 7.35 (d, *J* = 6.9 Hz, 1H), 7.27 (d, *J* = 1.5 Hz, 2H), 7.17 (d, *J* = 7.4 Hz, 1H), 7.13 – 7.06 (m, 1H), 2.24 (ddd, *J* = 12.2, 7.9, 3.4 Hz, 1H), 1.73 – 1.66 (m, 4H), 1.60 – 1.57 (m, 1H), 1.51 (d, *J* = 10.6 Hz, 2H), 1.19 – 1.07 (m, 3H). ^{13}C NMR (150 MHz, $CDCl_3$) δ 196.68 (s), 155.83 (s), 141.56 (s), 140.70 (s), 136.01 (s), 134.22 (s), 132.96 (s), 129.56 (s), 128.36 (s), 128.29 (s), 127.86 (s), 120.62 (s), 35.84 (s), 30.95 (s), 26.43 (s), 25.66 (s).

2-Cyclopentyl-3-phenyl-1*H*-inden-1-one (3r)[1]

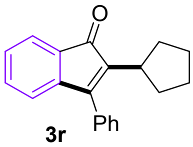

3r

The product purified by flash column chromatography on silica gel (PE/EA = 50/1) to afford the **3r** as a yellow solid (83% yield). ^{1}H NMR (600 MHz, $CDCl_3$) δ 7.43 (t, *J* = 7.4 Hz, 2H), 7.40 – 7.31 (m, 4H), 7.21 – 7.16 (m, 1H), 7.10 (t, *J* = 7.3 Hz, 1H), 6.84 (d, *J* = 7.2 Hz, 1H), 2.80 – 2.71 (m, 1H), 1.86 – 1.80 (m, 2H), 1.80 – 1.75 (m, 2H), 1.66 (ddd, *J* = 14.2, 9.8, 5.3 Hz, 2H), 1.50 – 1.45 (m, 2H). ^{13}C NMR (150 MHz, $CDCl_3$) δ 198.03 (s), 155.09 (s), 145.58 (s), 137.70 (s), 133.15, 133.04, 131.21 (s), 128.96 (s), 128.72 (s), 128.15, 128.02, 122.20 (s), 120.32 (s), 36.30 (s), 32.18 (s), 26.45 (s).

(*S*)-2-(*sec*-butyl)-3-phenyl-1*H*-inden-1-one (3s)[1]

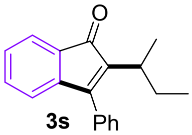

3s

The product purified by flash column chromatography on silica gel (PE/EA = 50/1) to afford the **3s** as a yellow oil (46% yield). ^{1}H NMR (600 MHz, $CDCl_3$) δ 7.50 (dd, *J* = 10.2, 4.6 Hz, 2H), 7.47 – 7.41 (m, 2H), 7.38 (dd, *J* = 5.2, 3.2 Hz, 2H), 7.30 – 7.23 (m, 1H), 7.21 – 7.15 (m, 1H), 6.88 (d, *J* = 7.2 Hz, 1H), 2.57 (dt, *J* = 8.5, 6.9 Hz, 1H), 1.81 – 1.72 (m, 1H), 1.59 – 1.51 (m, 1H), 1.23 (d, *J* = 7.0 Hz, 3H), 0.80 (t, *J* = 7.4 Hz, 3H). ^{13}C NMR (150 MHz, $CDCl_3$) δ 198.16 (s), 155.63 (s), 145.86 (s), 138.71 (s), 133.18, 133.06, 130.97 (s), 128.86 (s), 128.71 (s), 128.23 (s), 127.89 (s), 122.19 (s), 120.43 (s), 32.67 (s), 28.10 (s), 19.59 (s), 12.77 (s).

2-isopropyl-3-phenyl-1*H*-inden-1-one (3t)[1]

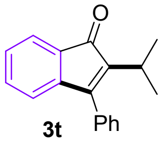

3t

The product purified by flash column chromatography on silica gel (PE/EA = 50/1) to afford the **3t** as a yellow oil (72% yield). ^{1}H NMR (600 MHz, $CDCl_3$) δ 7.43 (t, *J* = 7.4 Hz, 2H), 7.37 (dd, *J* = 11.8, 7.2 Hz, 2H), 7.34 – 7.29 (m, 2H), 7.19 (d, *J* = 5.4 Hz, 1H), 7.11 (t, *J* = 7.3 Hz, 1H), 6.83 (d, *J* = 7.2 Hz, 1H), 2.77 (dt, *J* = 14.0, 7.0 Hz, 1H), 1.18 (s, 3H), 1.17 (s, 3H). ^{13}C NMR (150

MHz, $CDCl_3$) δ 197.07 (s), 153.27 (s), 144.71 (s), 138.66 (s), 132.10, 131.97, 130.00 (s), 127.90 (s), 127.67 (s), 127.19 (s), 126.89 (s), 121.14 (s), 119.46 (s), 24.47 (s), 20.41 (s).

5. NMR spectra of compounds

^1H NMR of **3a** in $CDCl_3$ (600 MHz, $CDCl_3$)

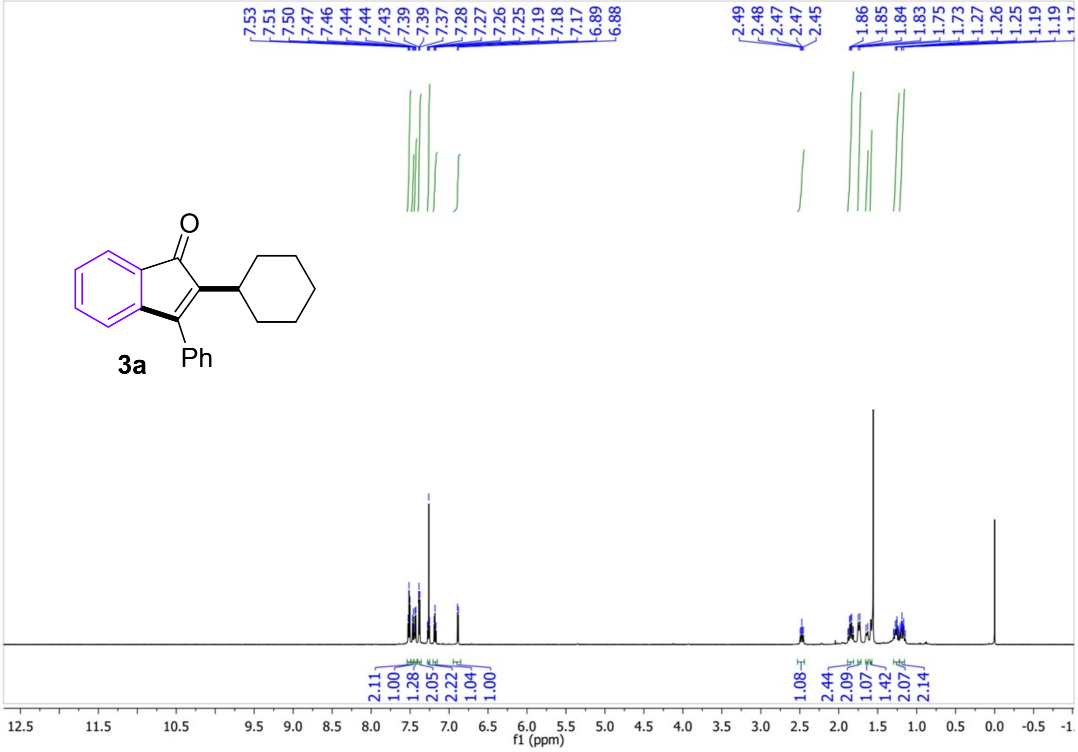

^{13}C NMR of **3a** in $CDCl_3$ (150 MHz, $CDCl_3$)

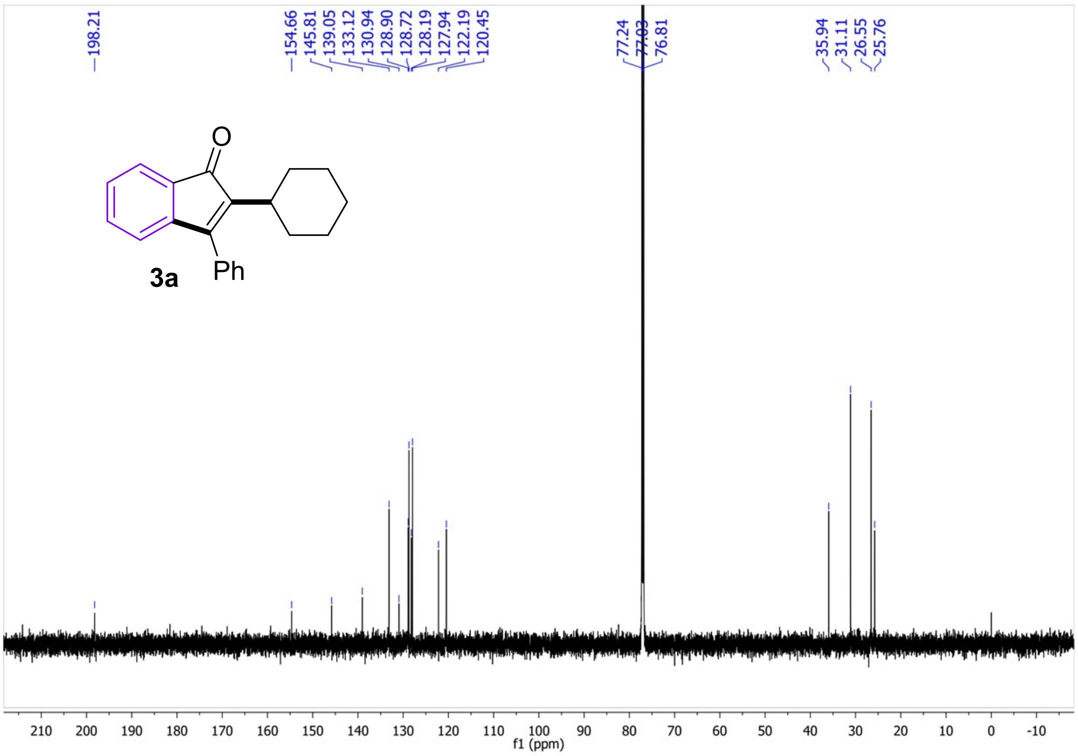

^{1}H NMR of **3b** in $CDCl_3$ (600 MHz, $CDCl_3$)

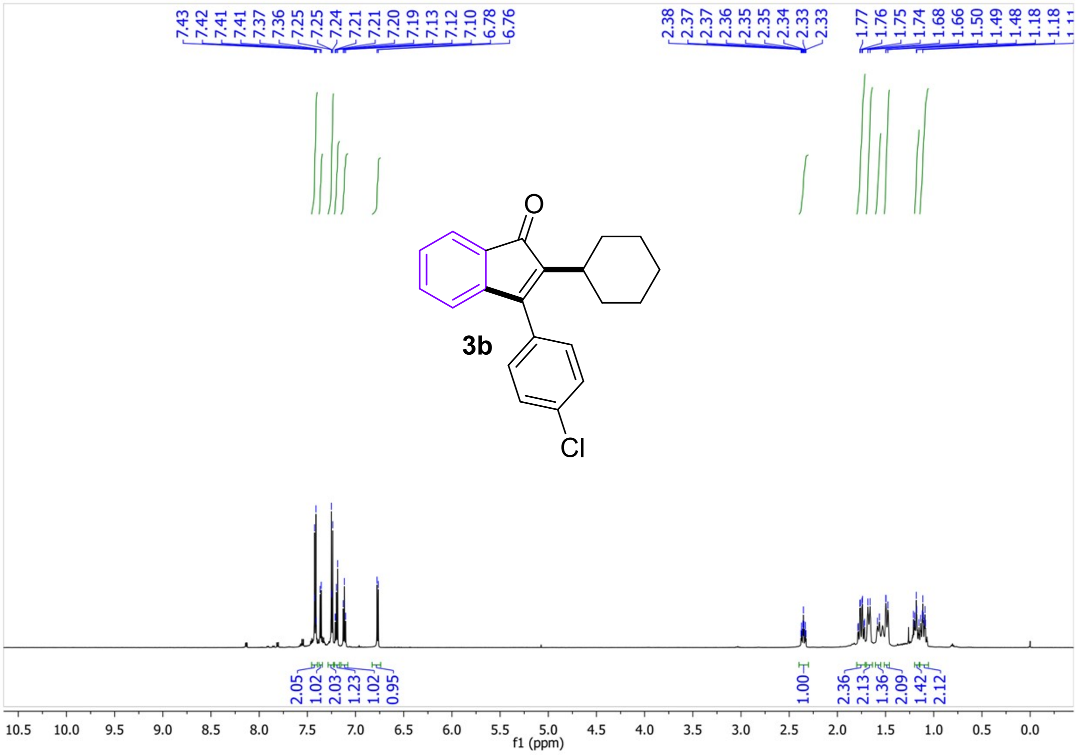

^{13}C NMR of **3b** in $CDCl_3$ (150 MHz, $CDCl_3$)

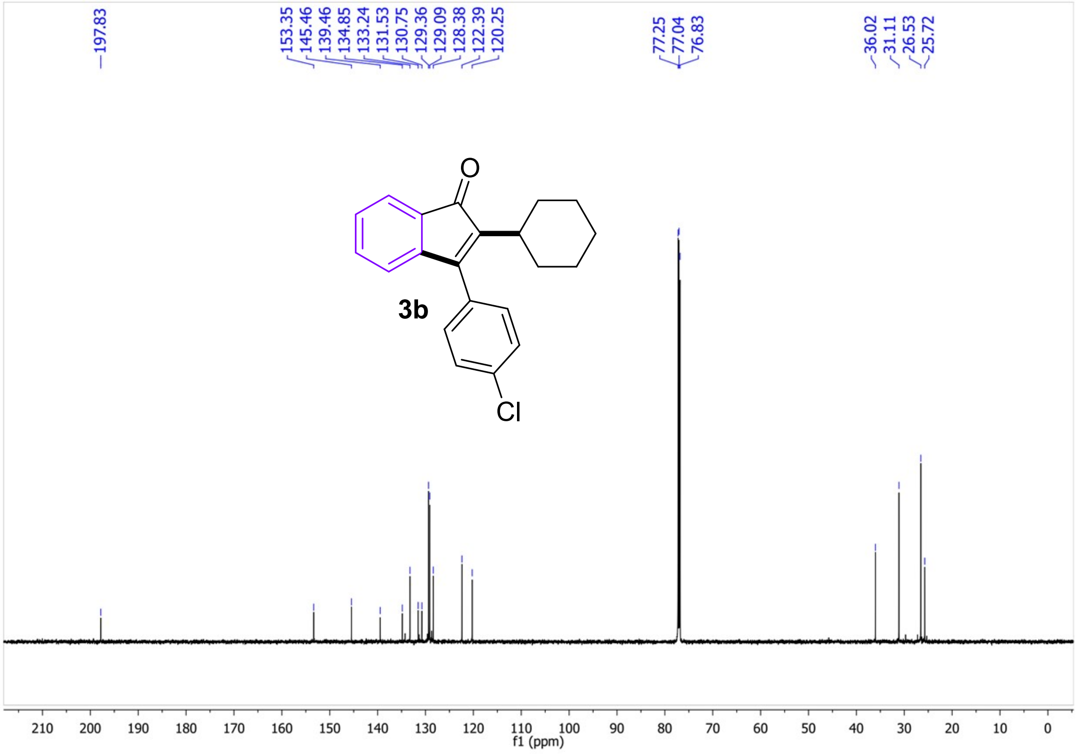

^{1}H NMR of **3c** in $CDCl_3$ (600 MHz, $CDCl_3$)

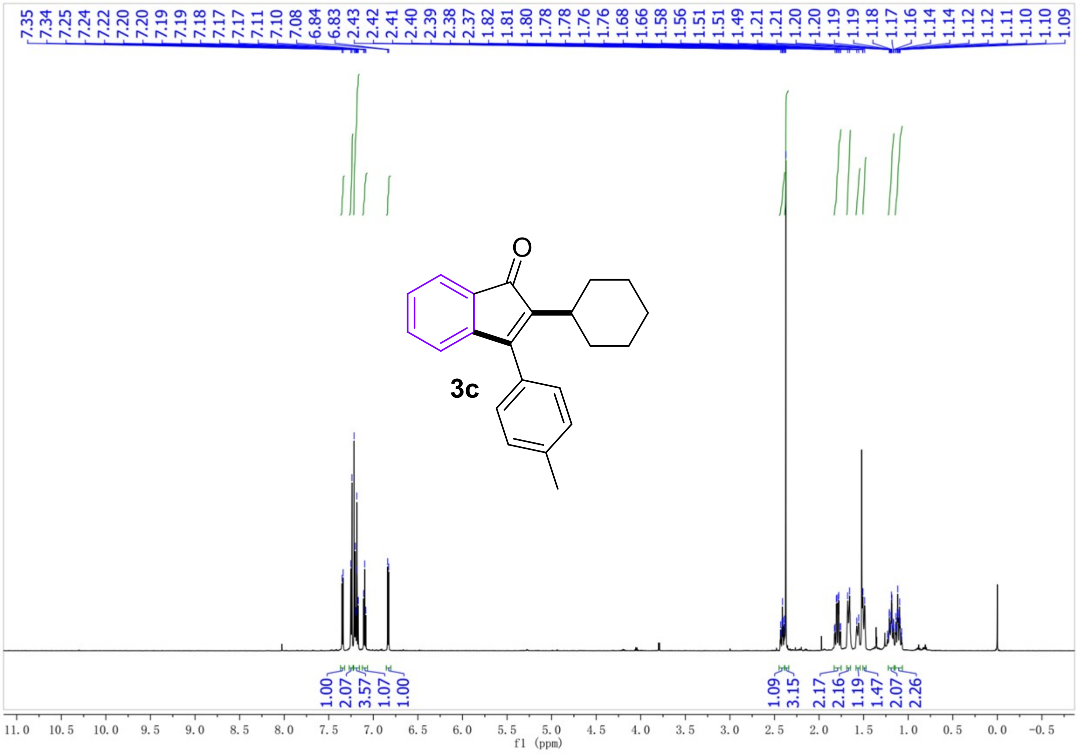

^{13}C NMR of **3c** in $CDCl_3$ (150 MHz, $CDCl_3$)

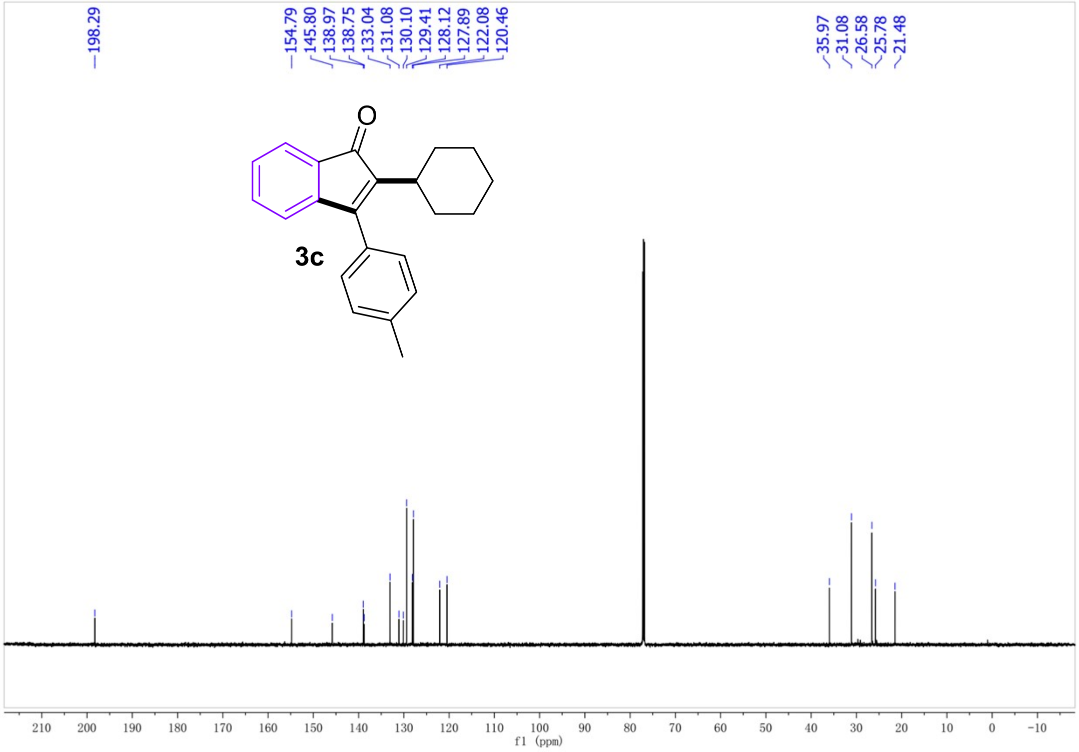

^{1}H NMR of **3d** in $CDCl_3$ (600 MHz, $CDCl_3$)

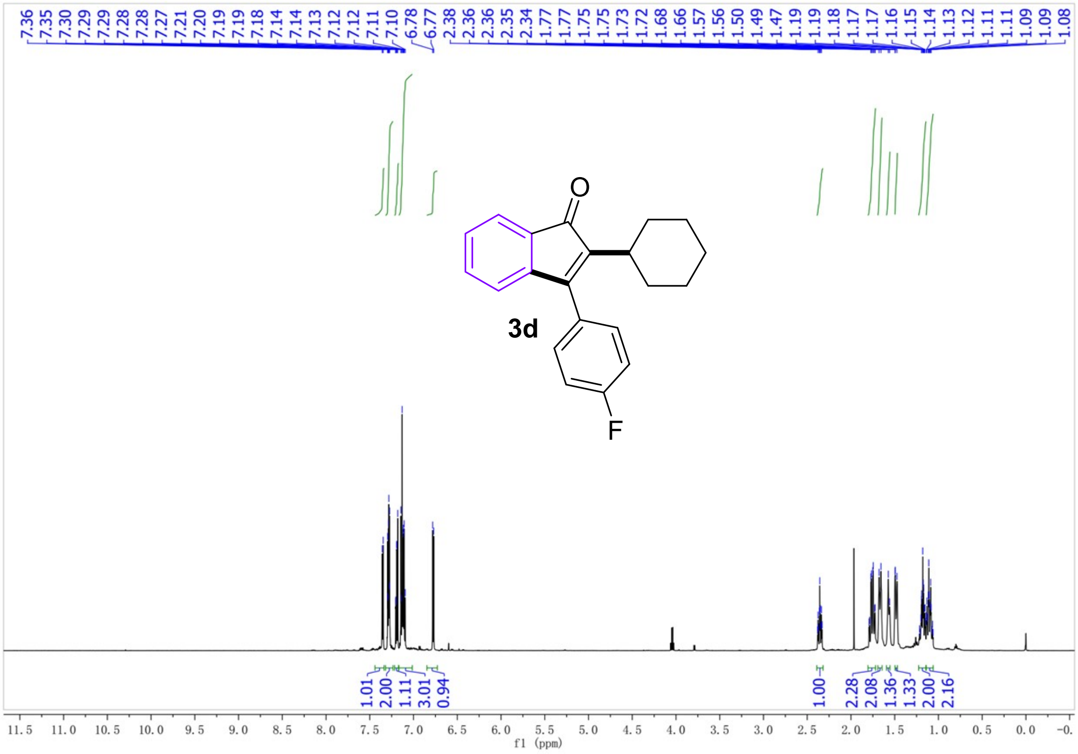

^{13}C NMR of **3d** in $CDCl_3$ (150 MHz, $CDCl_3$)

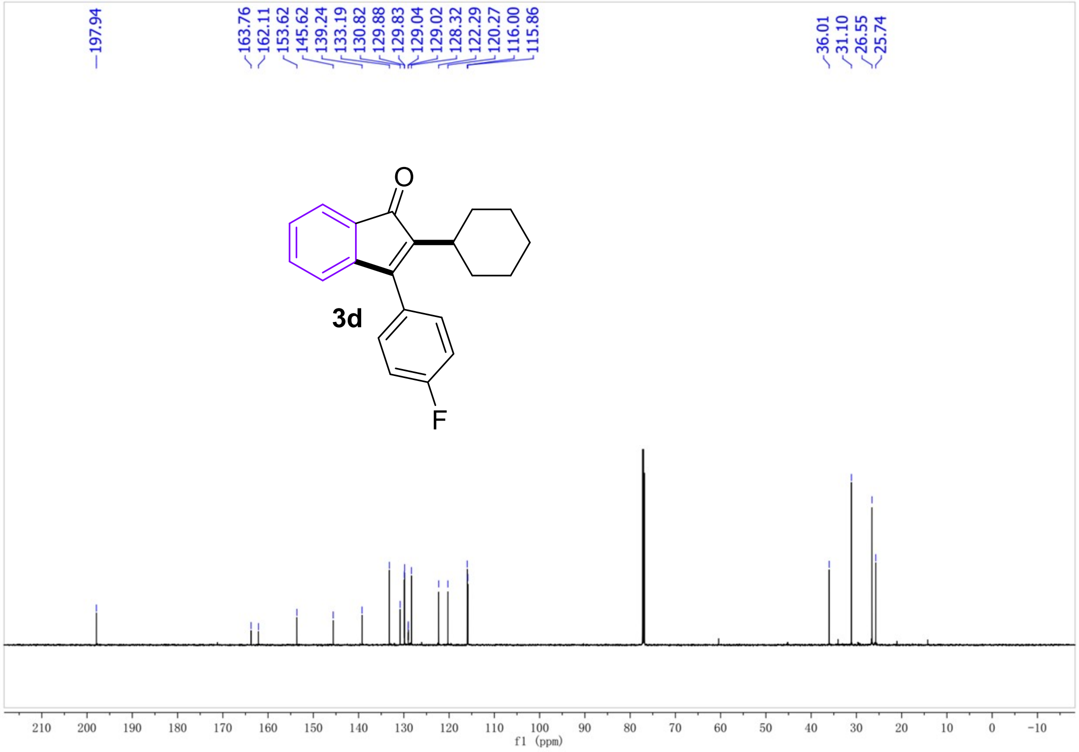

^{1}H NMR of **3e** in $CDCl_3$ (600 MHz, $CDCl_3$)

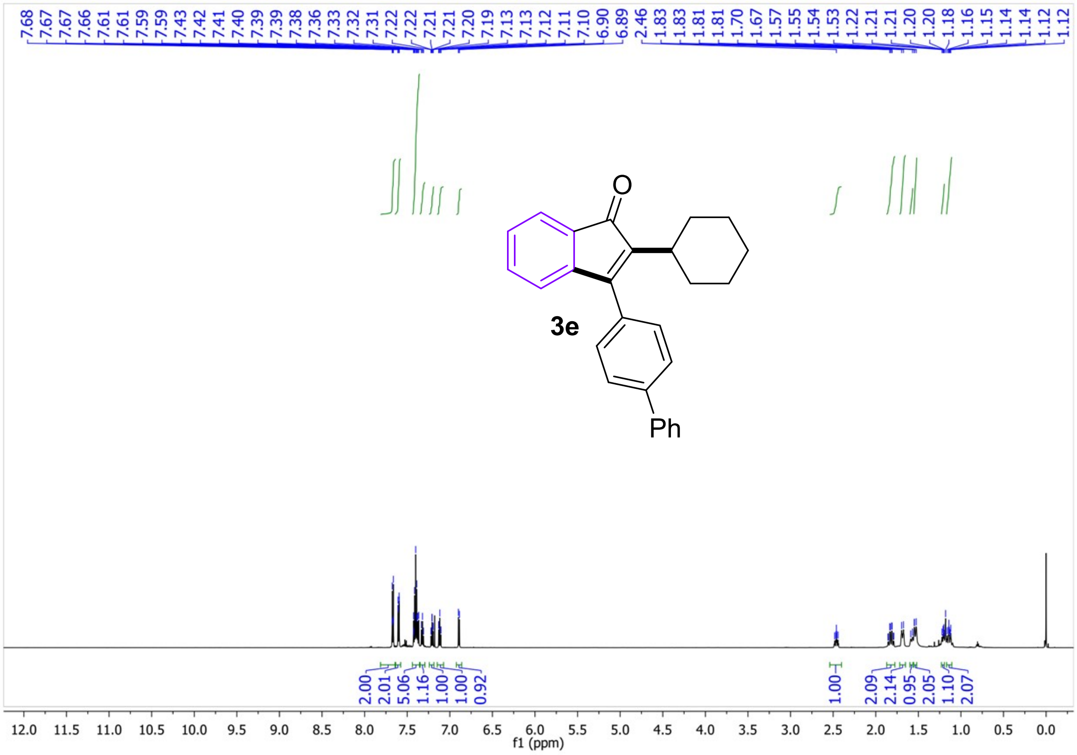

^{13}C NMR of **3e** in $CDCl_3$ (150 MHz, $CDCl_3$)

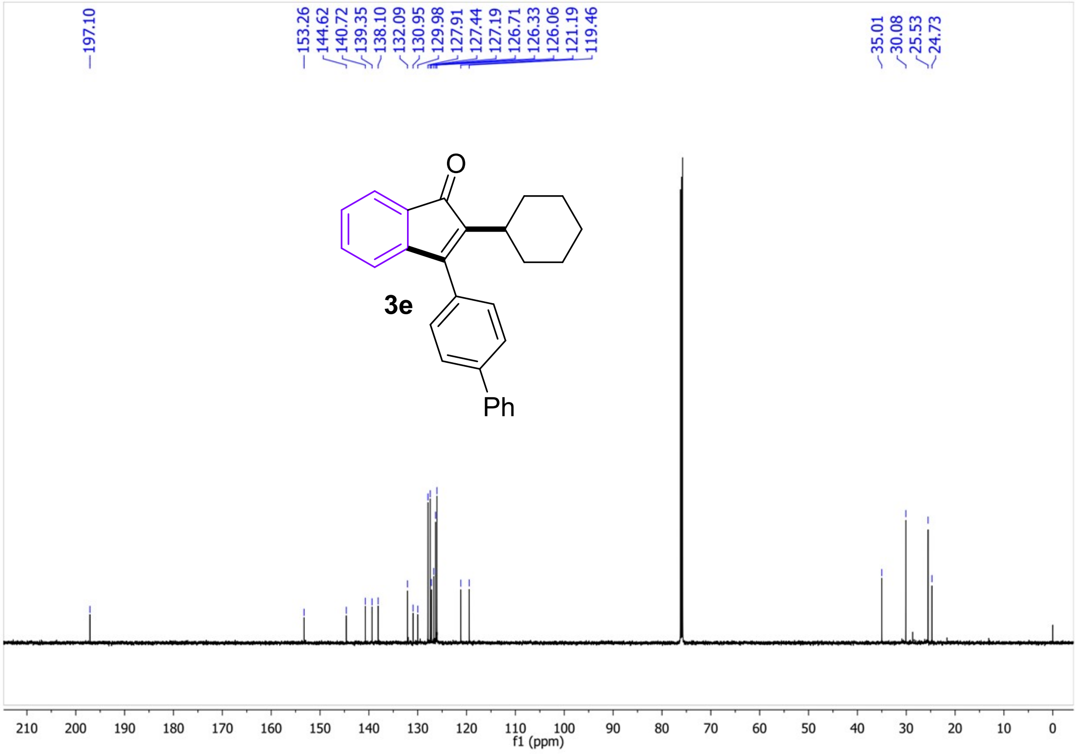

^1H NMR of **3f** in $CDCl_3$ (600 MHz, $CDCl_3$)

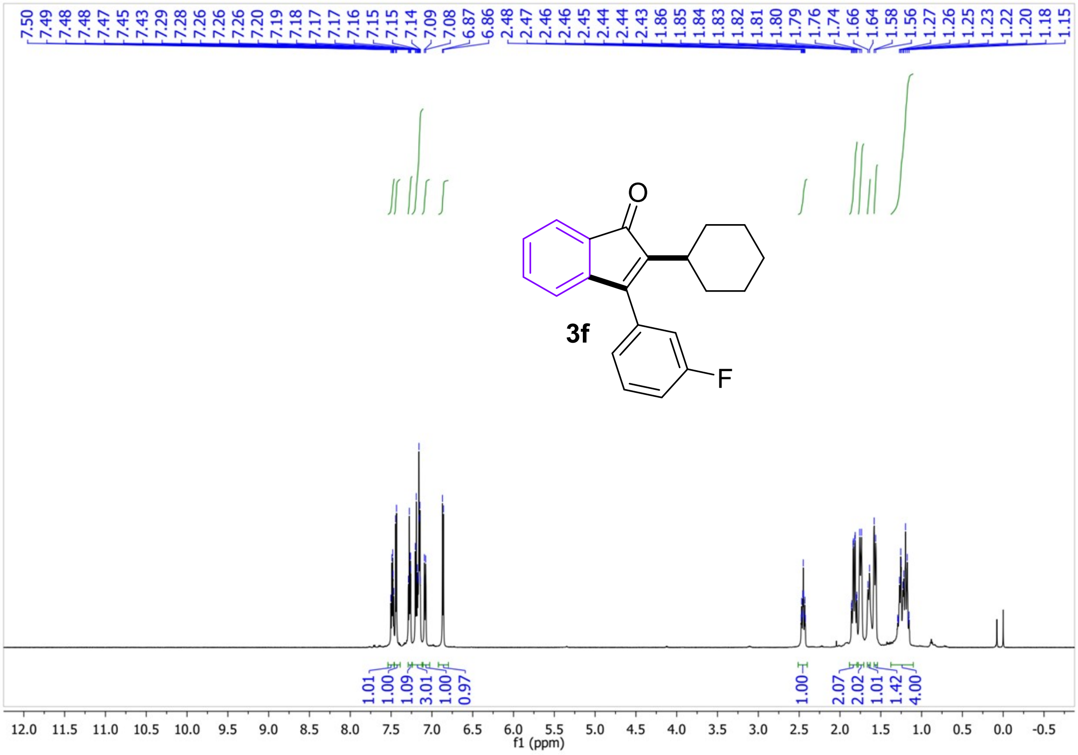

^{13}C NMR of **3f** in $CDCl_3$ (150 MHz, $CDCl_3$)

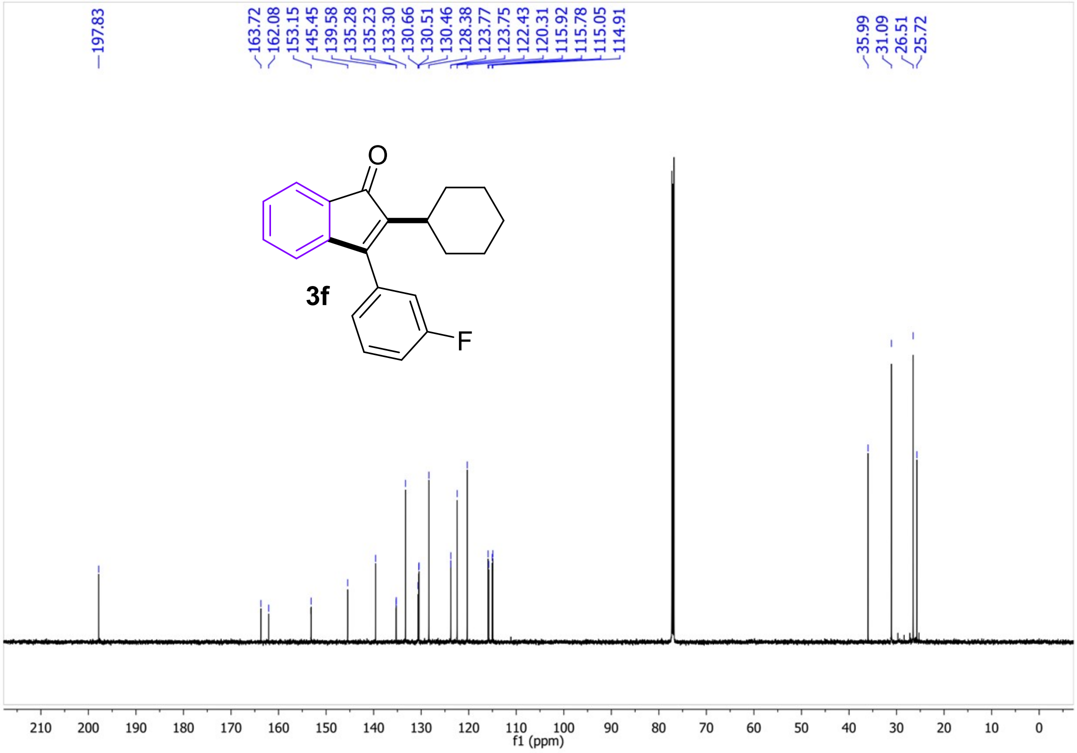

^{1}H NMR of **3g** in $CDCl_3$ (600 MHz, $CDCl_3$)

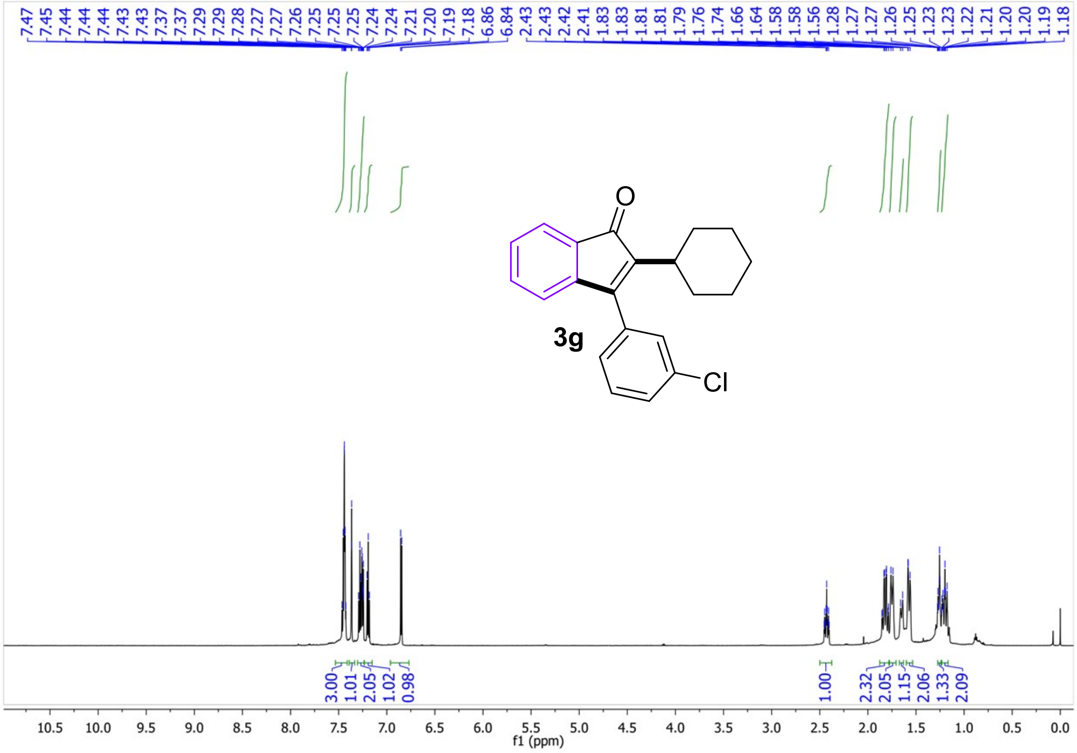

^{13}C NMR of **3g** in $CDCl_3$ (150 MHz, $CDCl_3$)

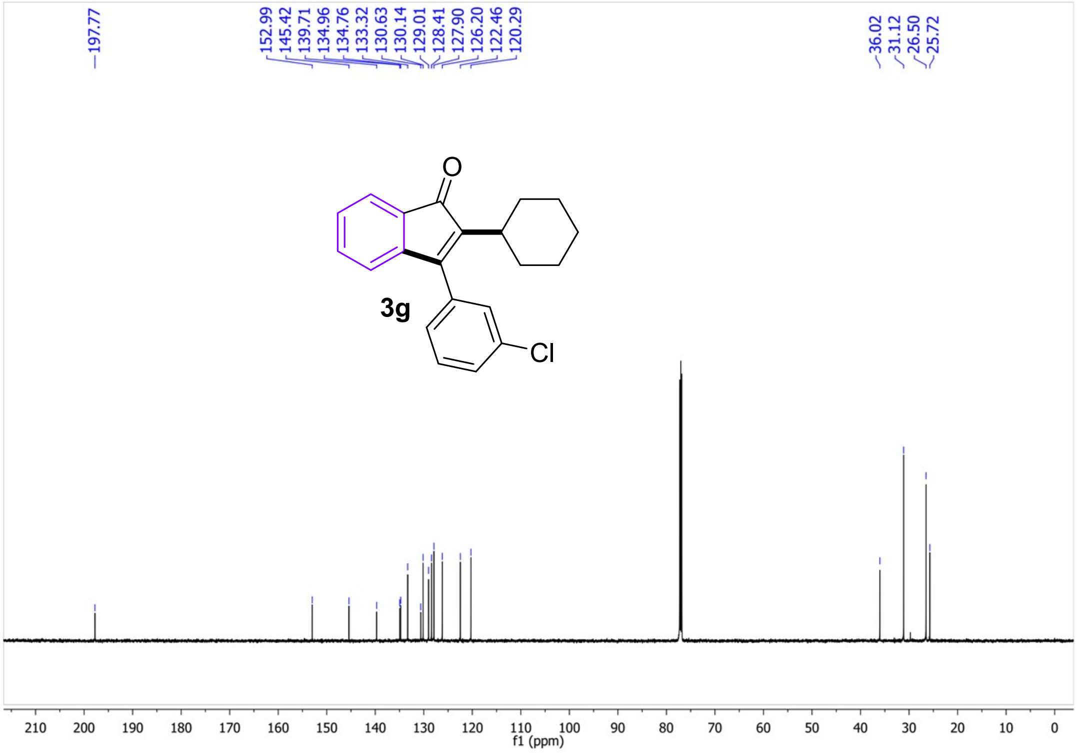

^{1}H NMR of **3h** in $CDCl_3$ (600 MHz, $CDCl_3$)

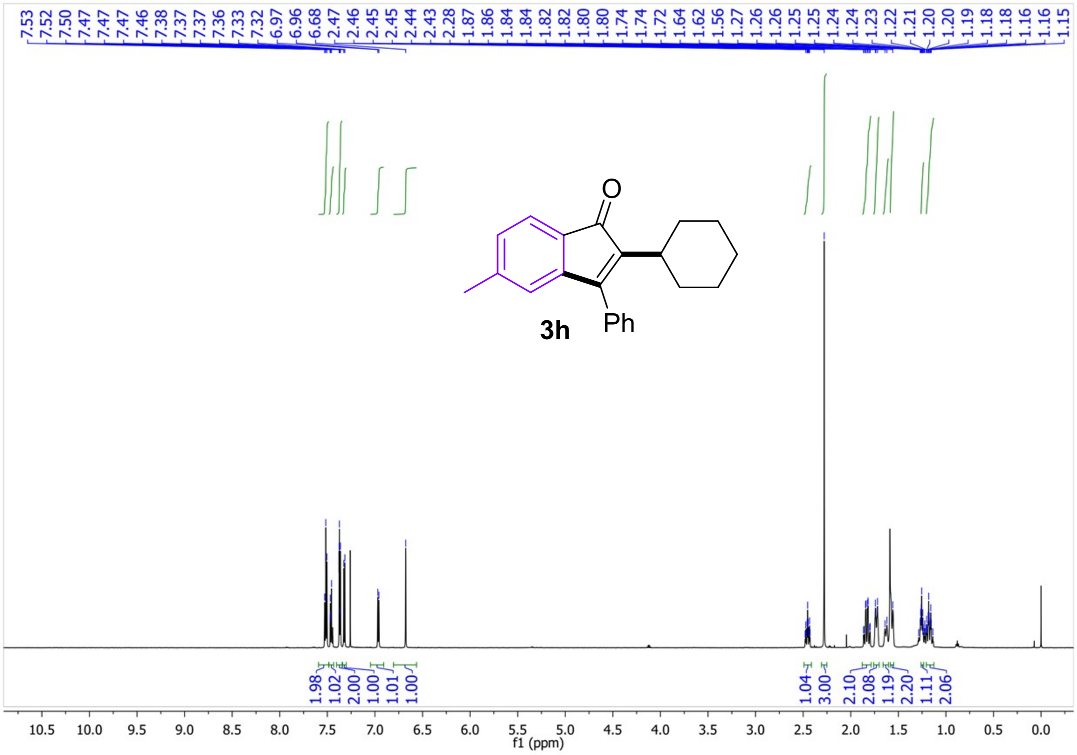

^{13}C NMR of **3h** in $CDCl_3$ (150 MHz, $CDCl_3$)

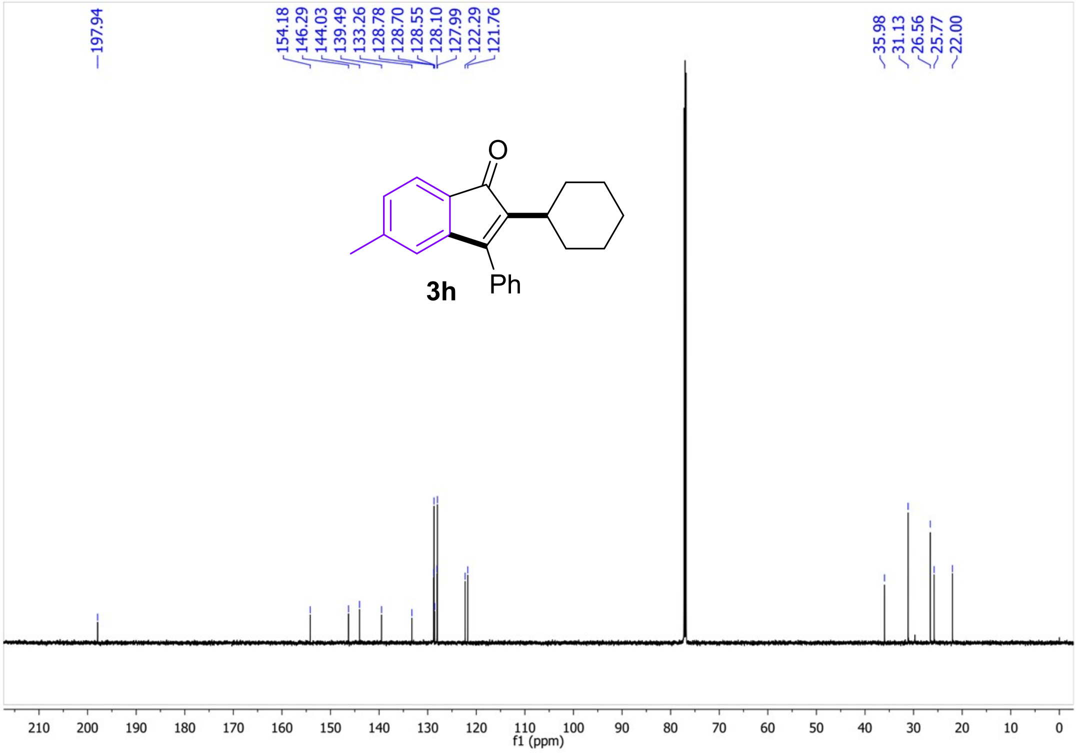

^{1}H NMR of **3i** in $CDCl_3$ (600 MHz, $CDCl_3$)

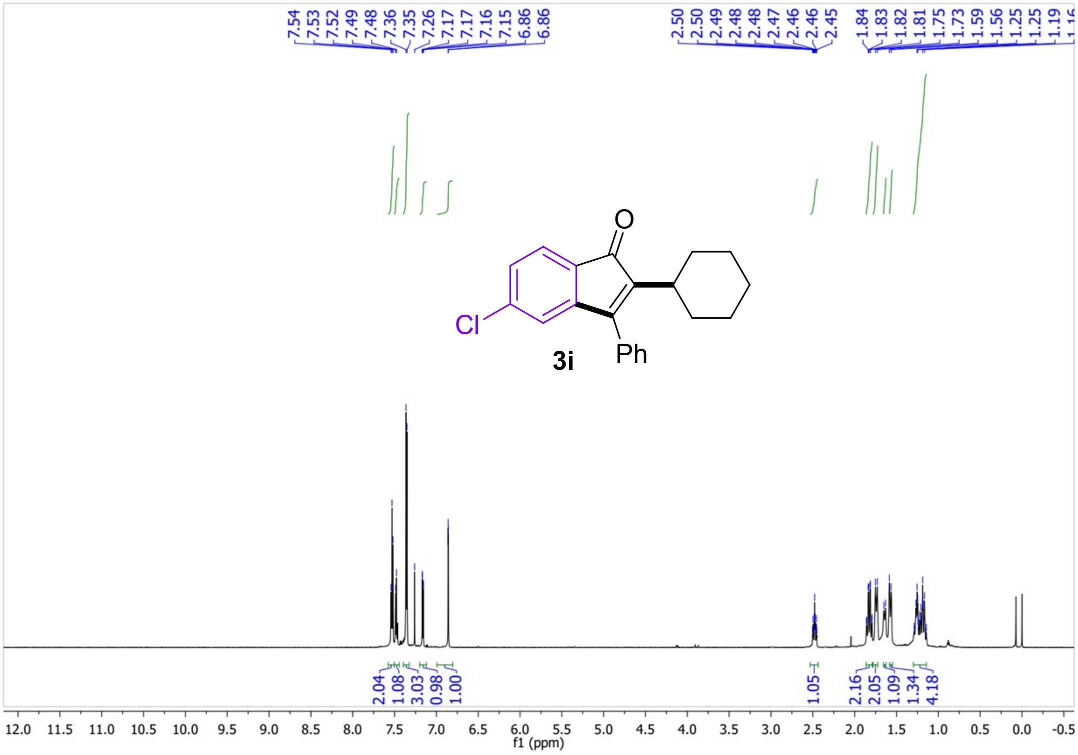

^{13}C NMR of **3i** in $CDCl_3$ (150 MHz, $CDCl_3$)

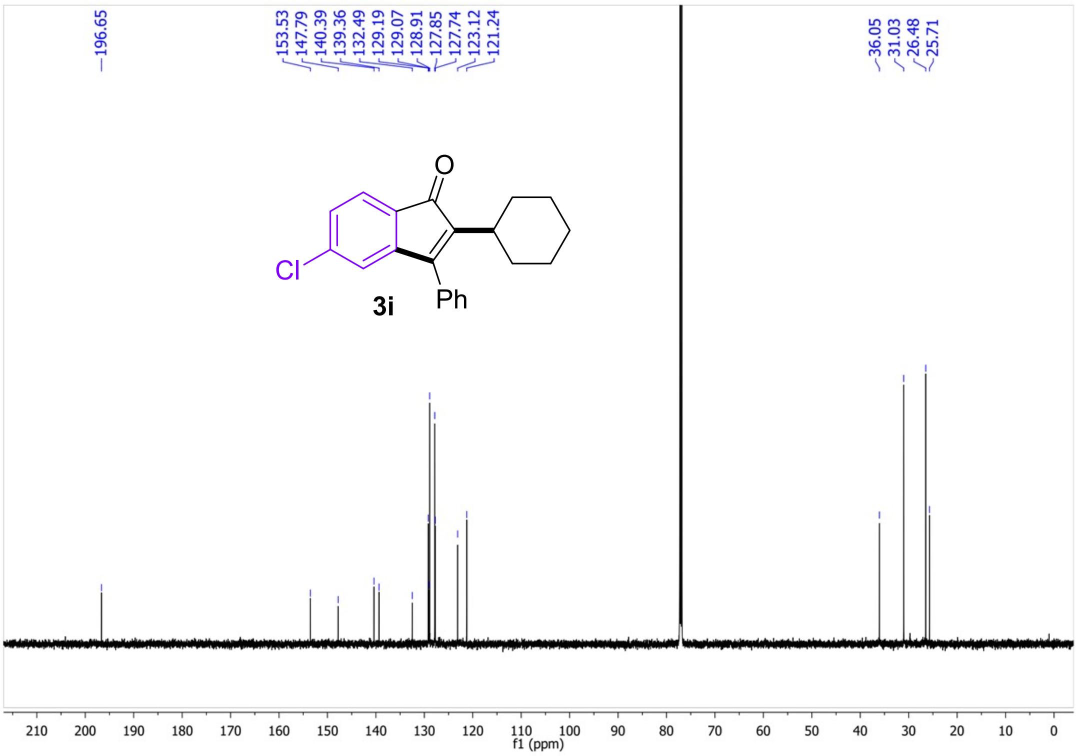

^{1}H NMR of **3j** in $CDCl_3$ (600 MHz, $CDCl_3$)

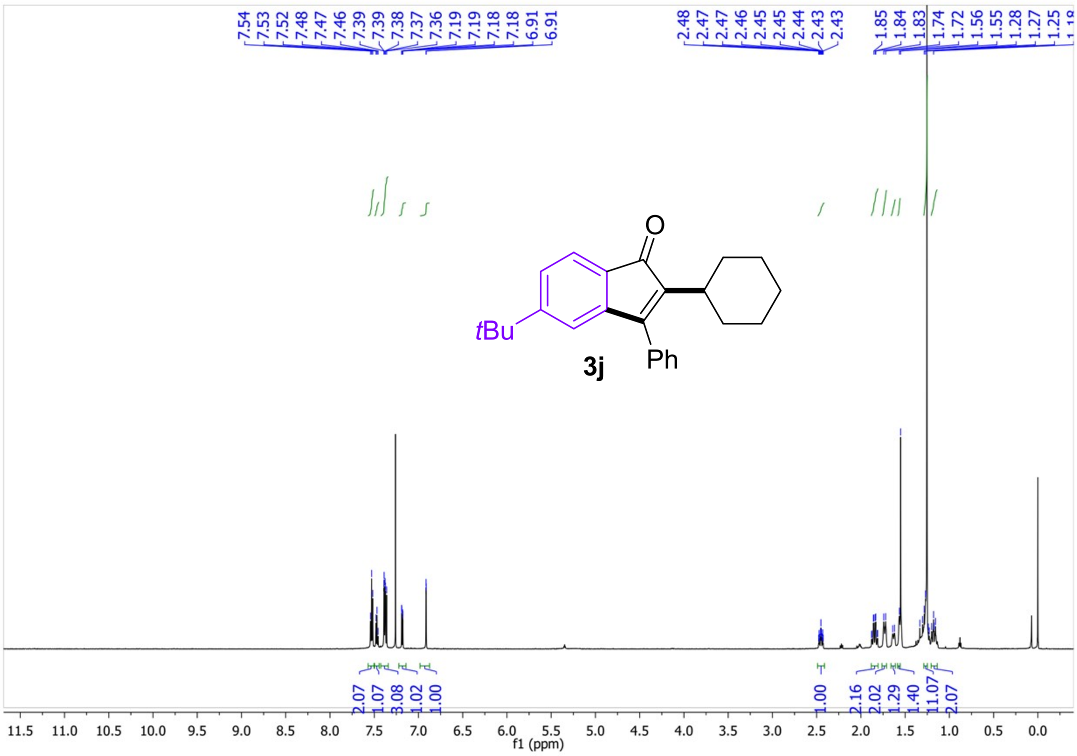

^{13}C NMR of **3j** in $CDCl_3$ (150 MHz, $CDCl_3$)

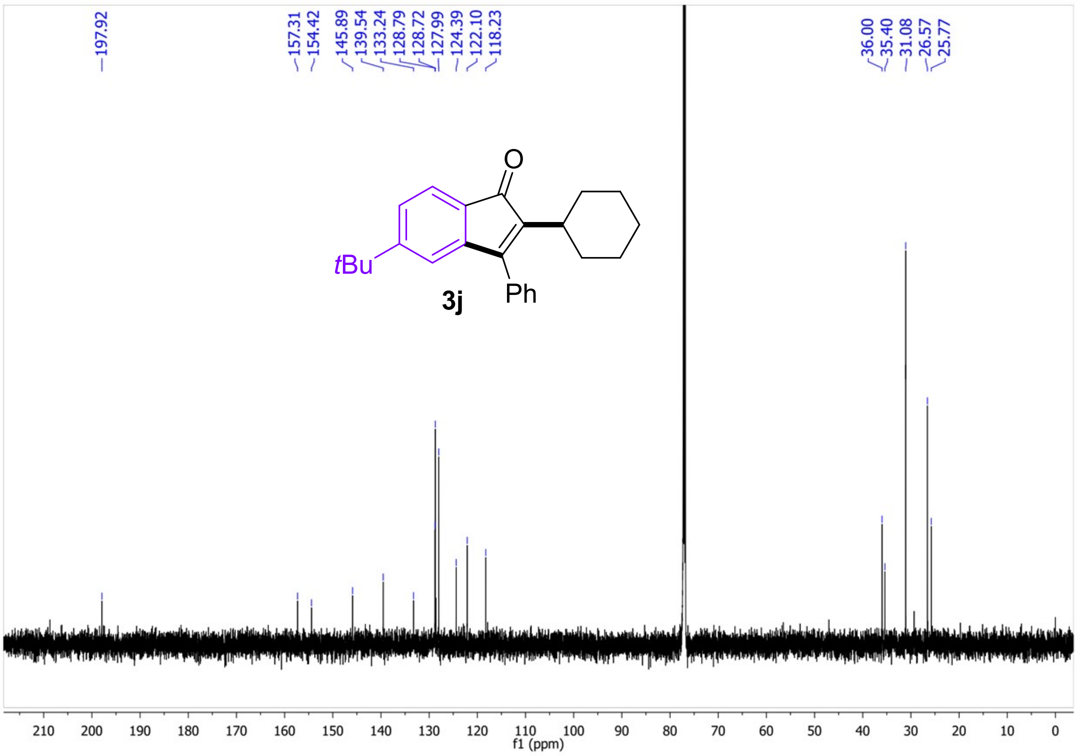

^{1}H NMR of **3k** in $CDCl_3$ (600 MHz, $CDCl_3$)

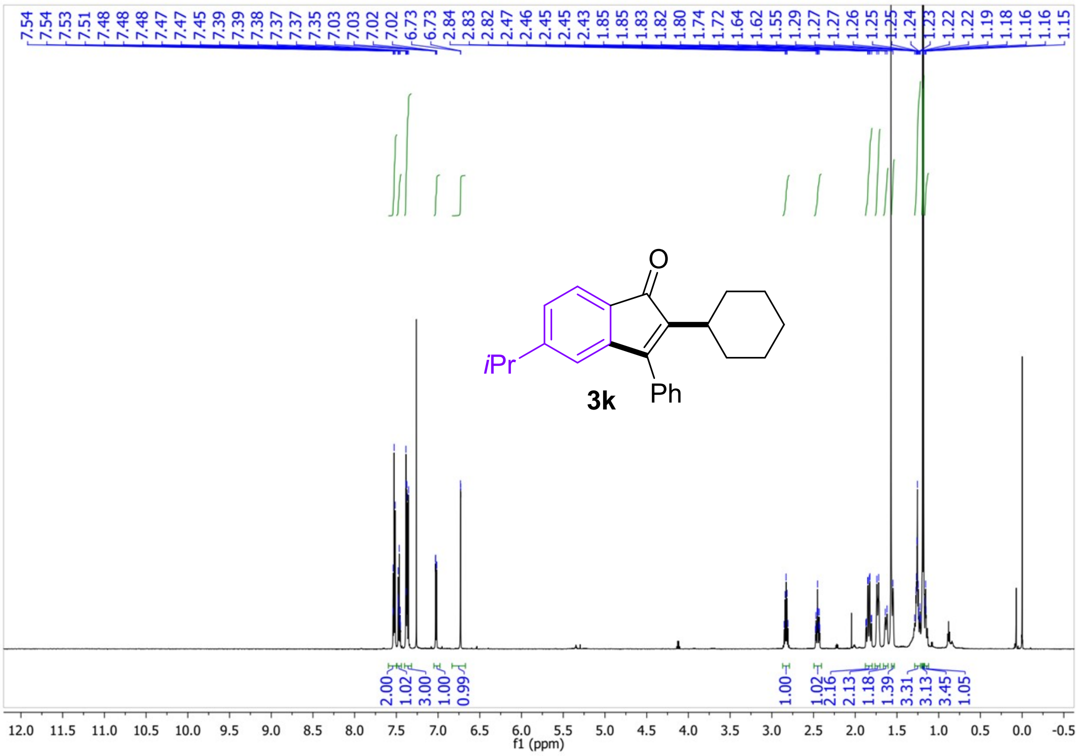

^{13}C NMR of **3k** in $CDCl_3$ (150 MHz, $CDCl_3$)

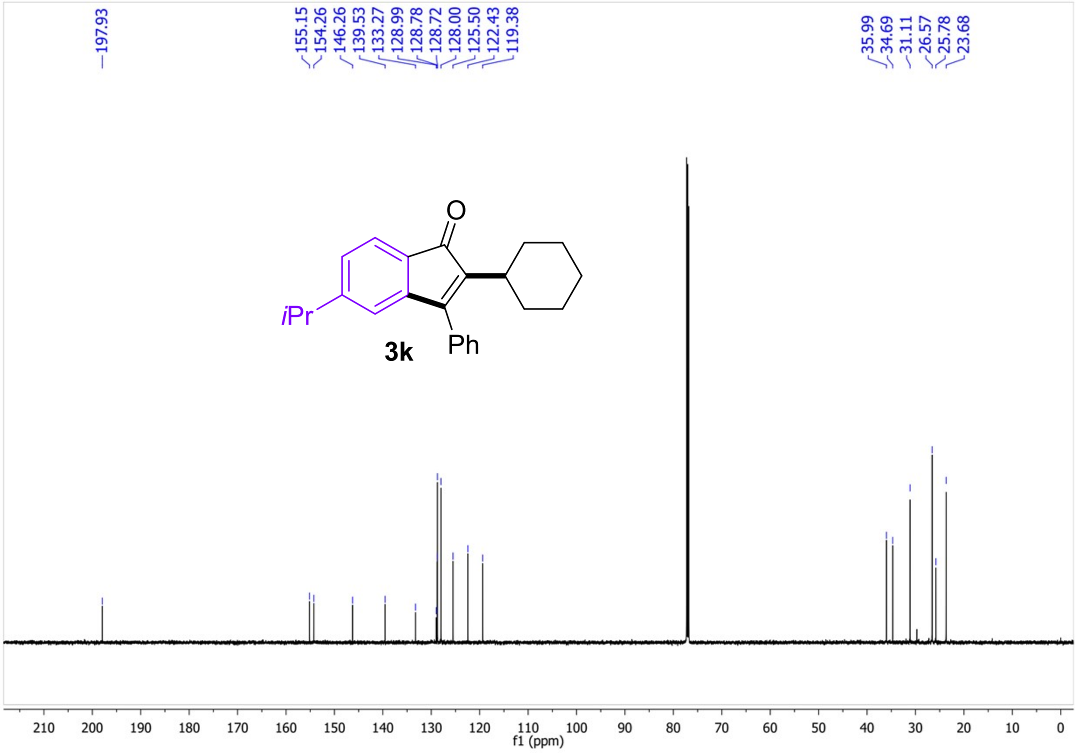

^{1}H NMR of **3l** in $CDCl_3$ (600 MHz, $CDCl_3$)

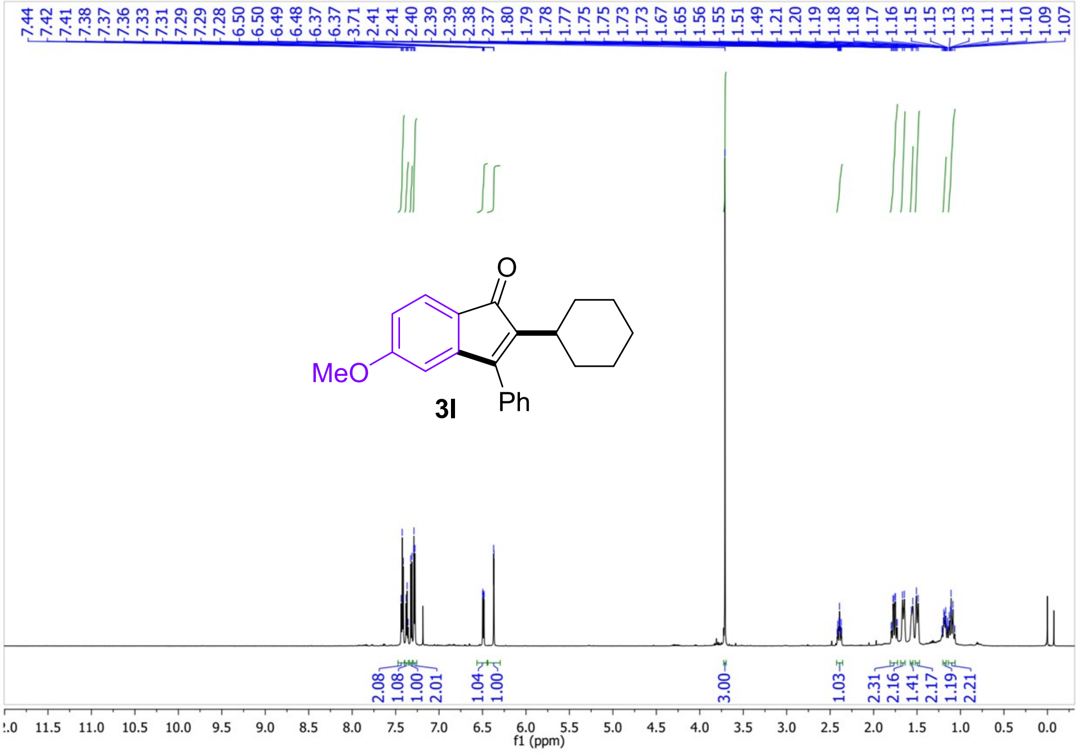

^{13}C NMR of **3l** in $CDCl_3$ (150 MHz, $CDCl_3$)

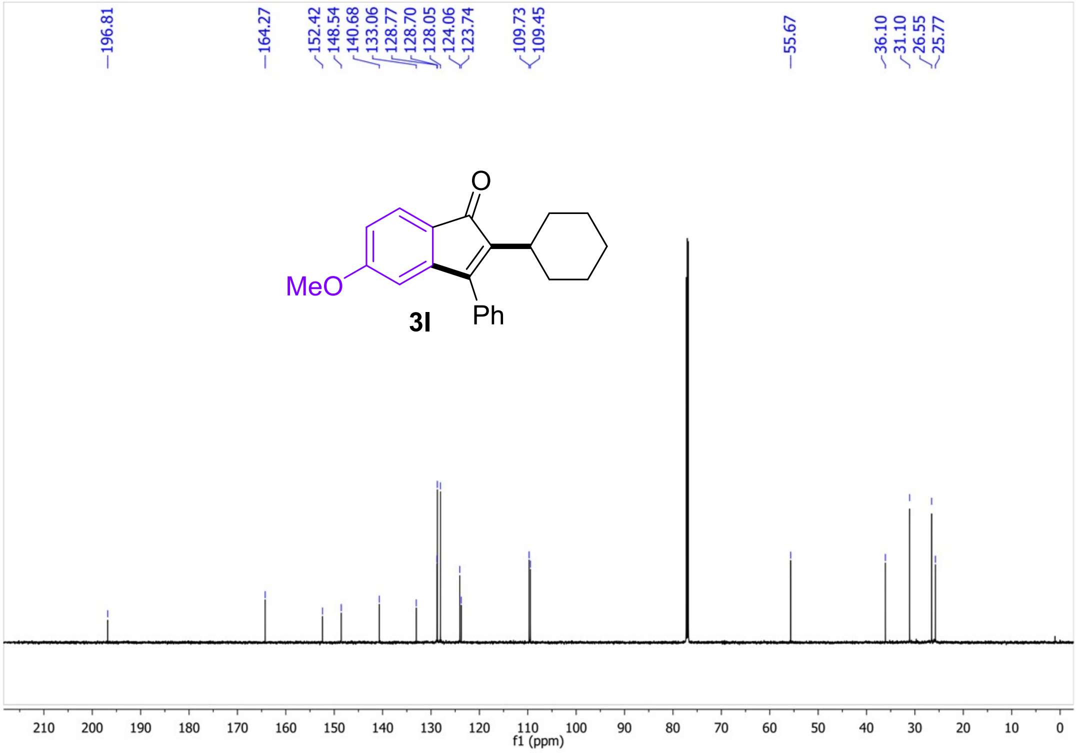

^{1}H NMR of **3m** in $CDCl_3$ (600 MHz, $CDCl_3$)

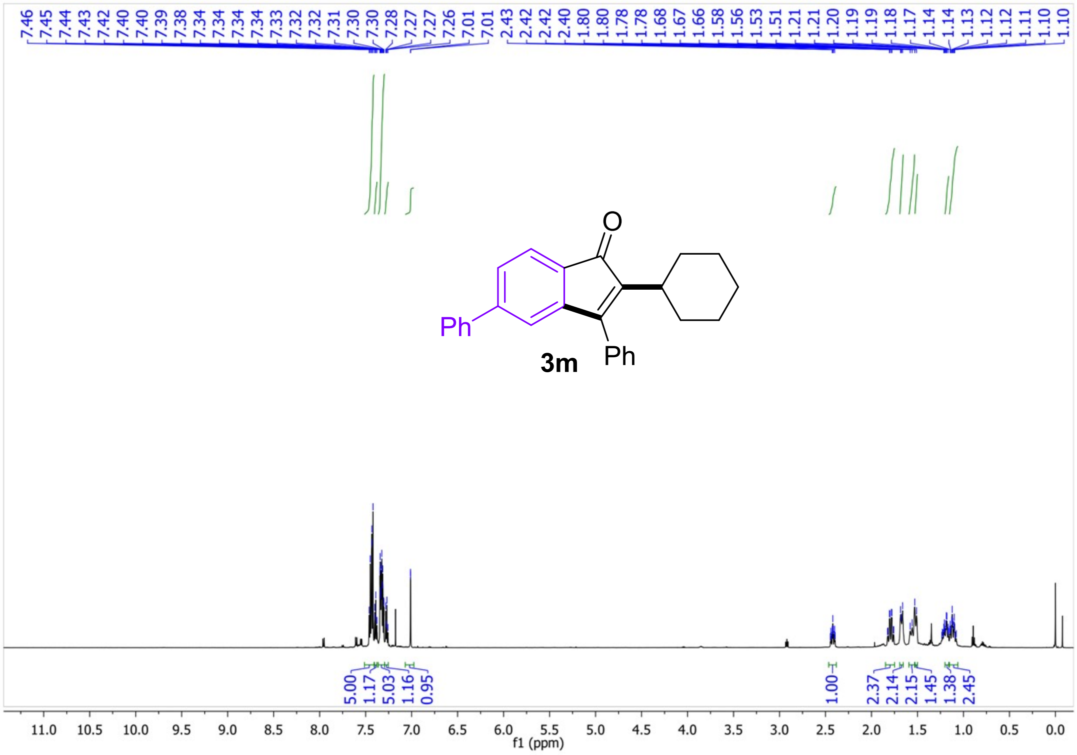

^{13}C NMR of **3m** in $CDCl_3$ (150 MHz, $CDCl_3$)

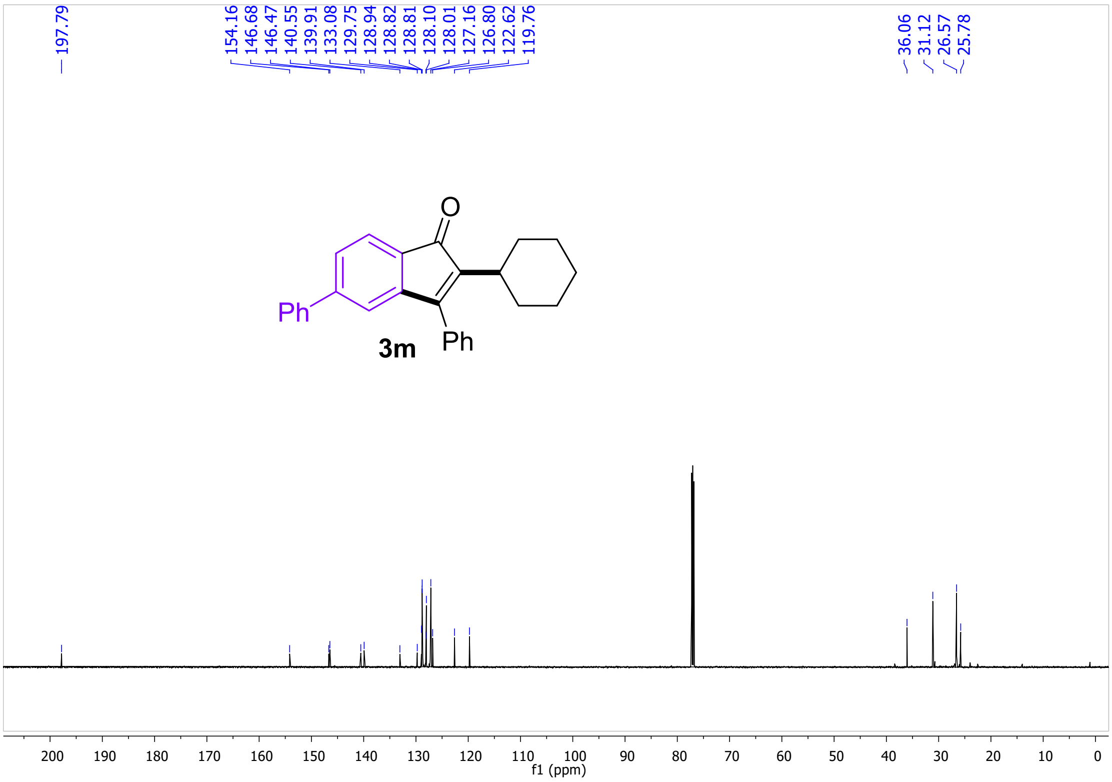

^{1}H NMR of **3o** in $CDCl_3$ (600 MHz, $CDCl_3$)

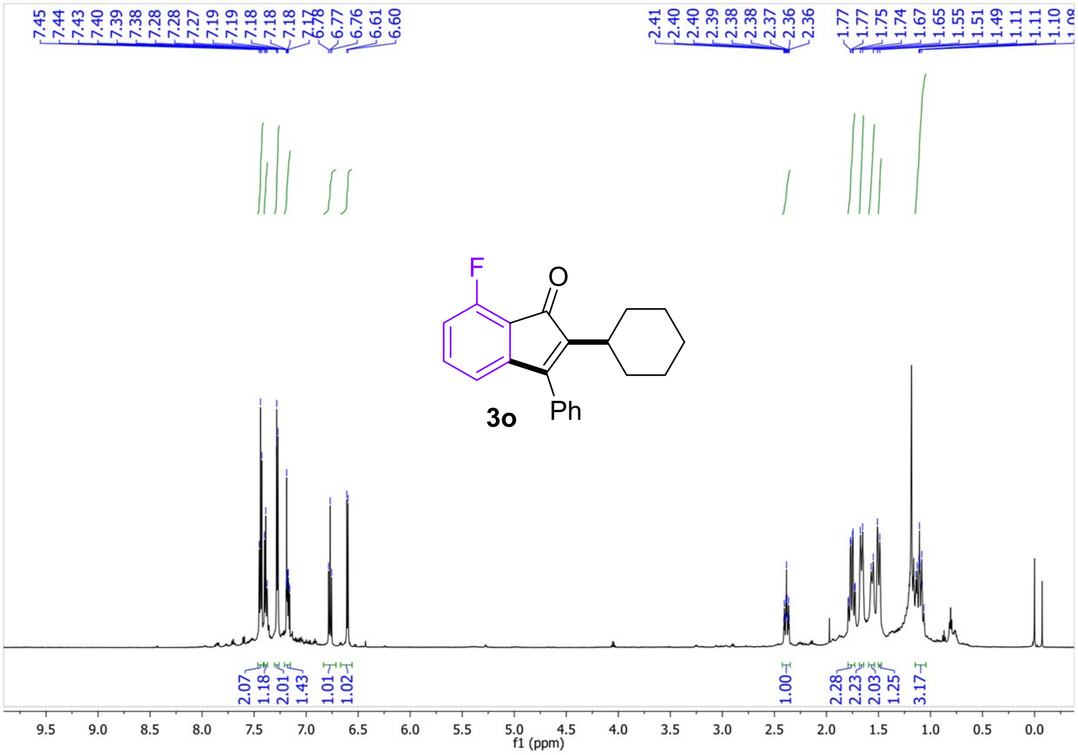

^{13}C NMR of **3o** in $CDCl_3$ (150 MHz, $CDCl_3$)

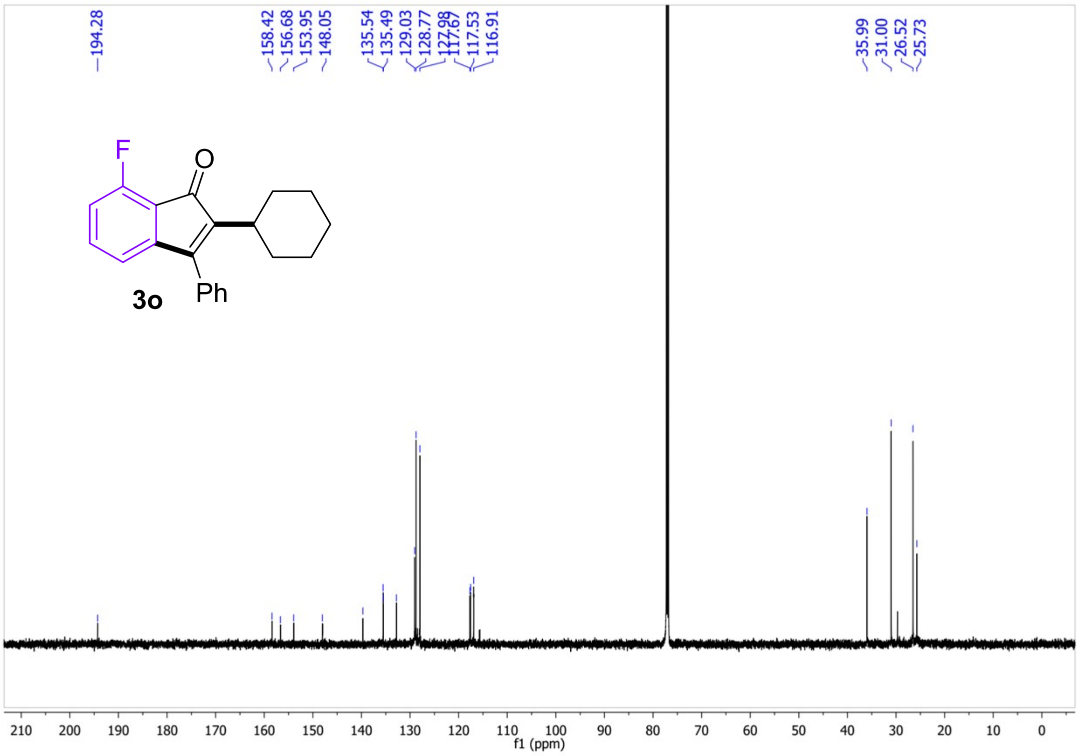

^{1}H NMR of **3p** in $CDCl_3$ (600 MHz, $CDCl_3$)

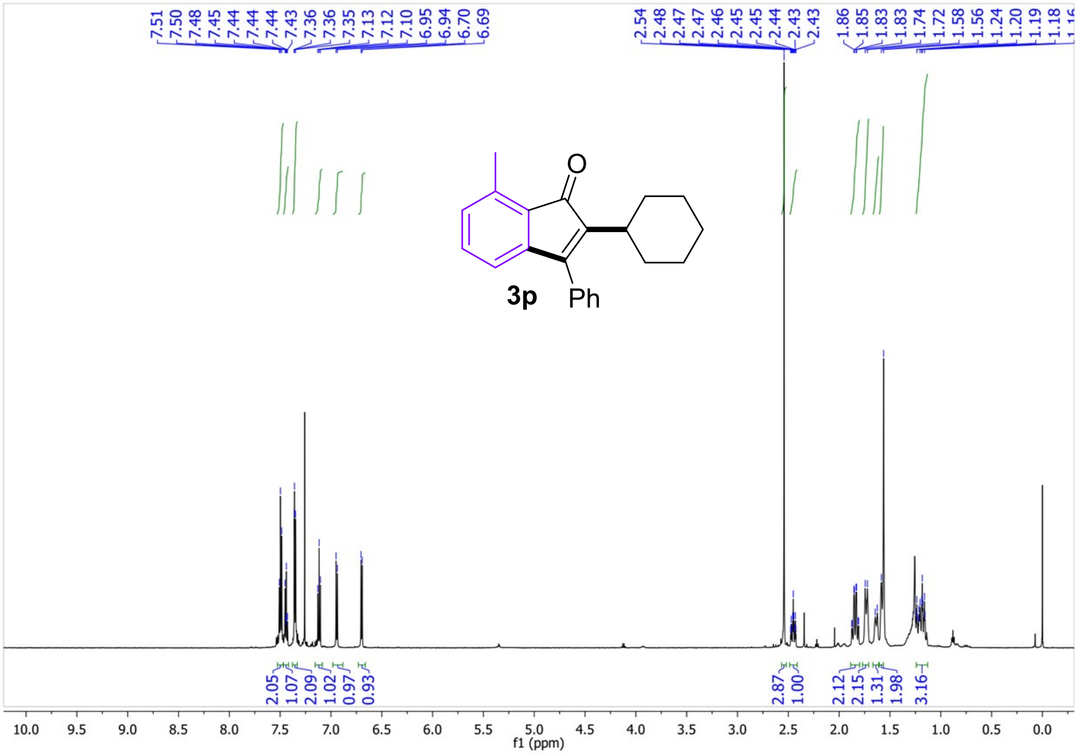

^{13}C NMR of **3p** in $CDCl_3$ (150 MHz, $CDCl_3$)

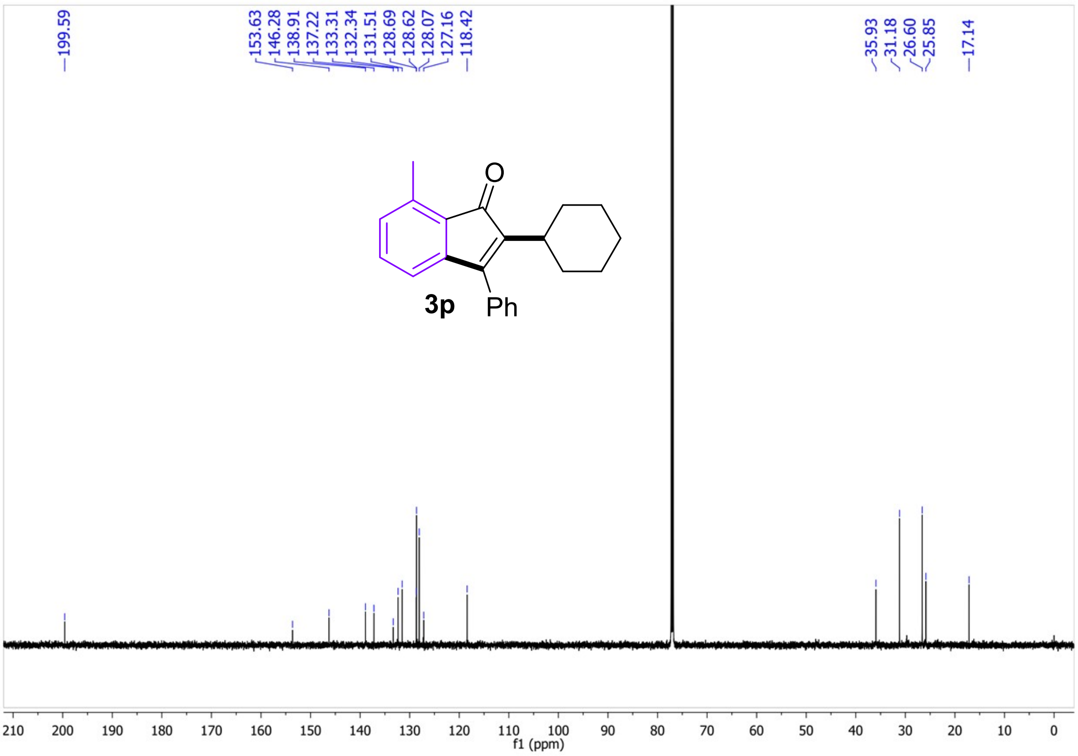

^{1}H NMR of **3q** in $CDCl_3$ (600 MHz, $CDCl_3$)

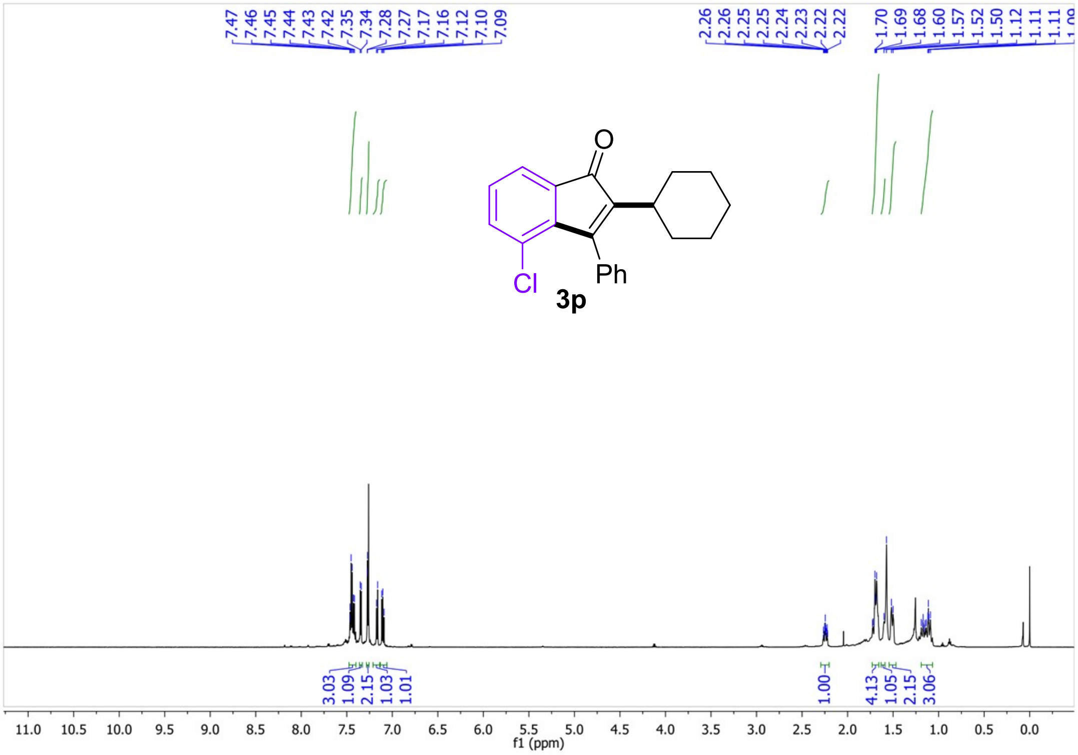

^{13}C NMR of **3q** in $CDCl_3$ (150 MHz, $CDCl_3$)

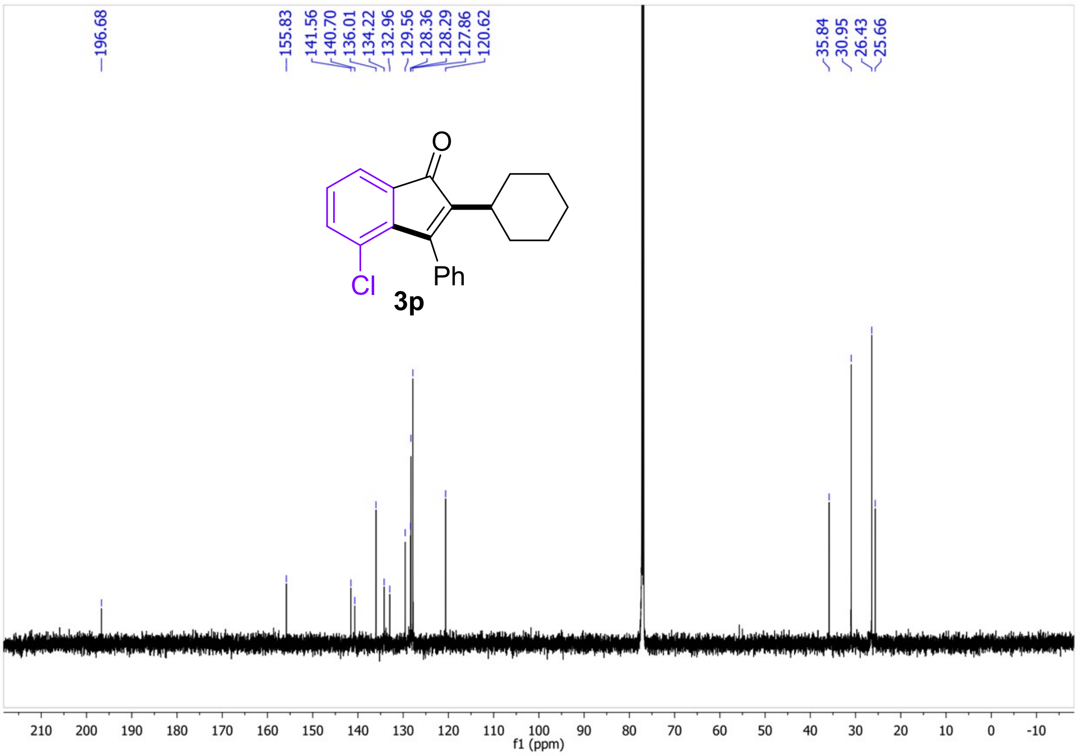

^{1}H NMR of **3r** in $CDCl_3$ (600 MHz, $CDCl_3$)

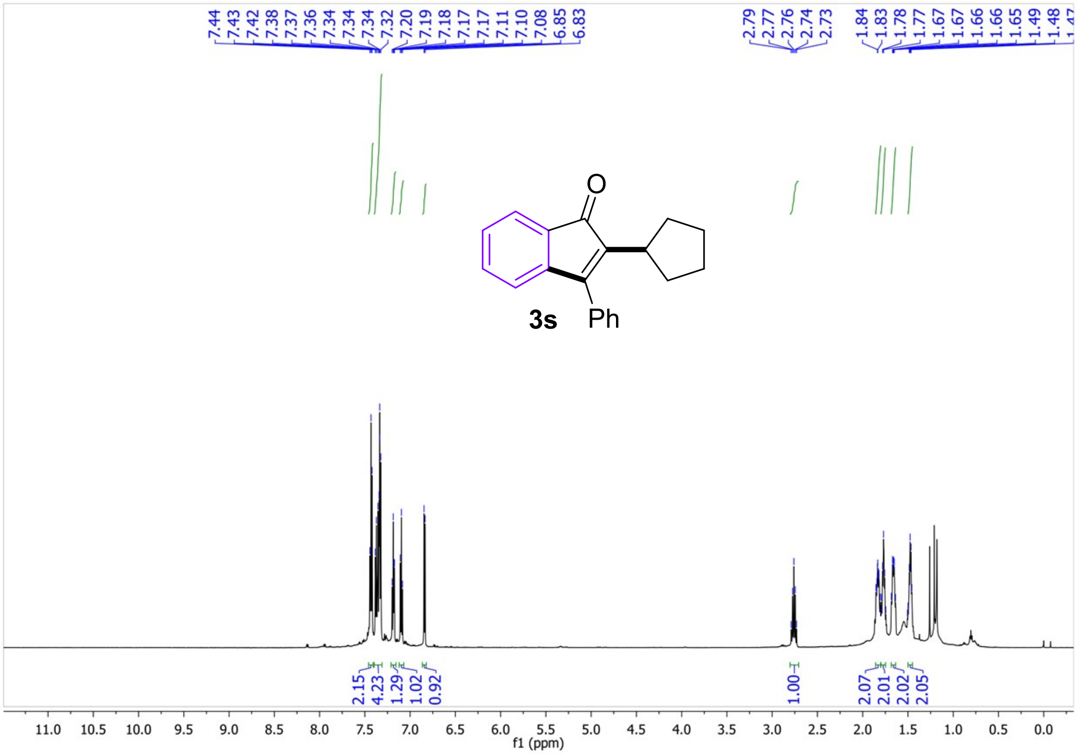

^{13}C NMR of **3r** in $CDCl_3$ (150 MHz, $CDCl_3$)

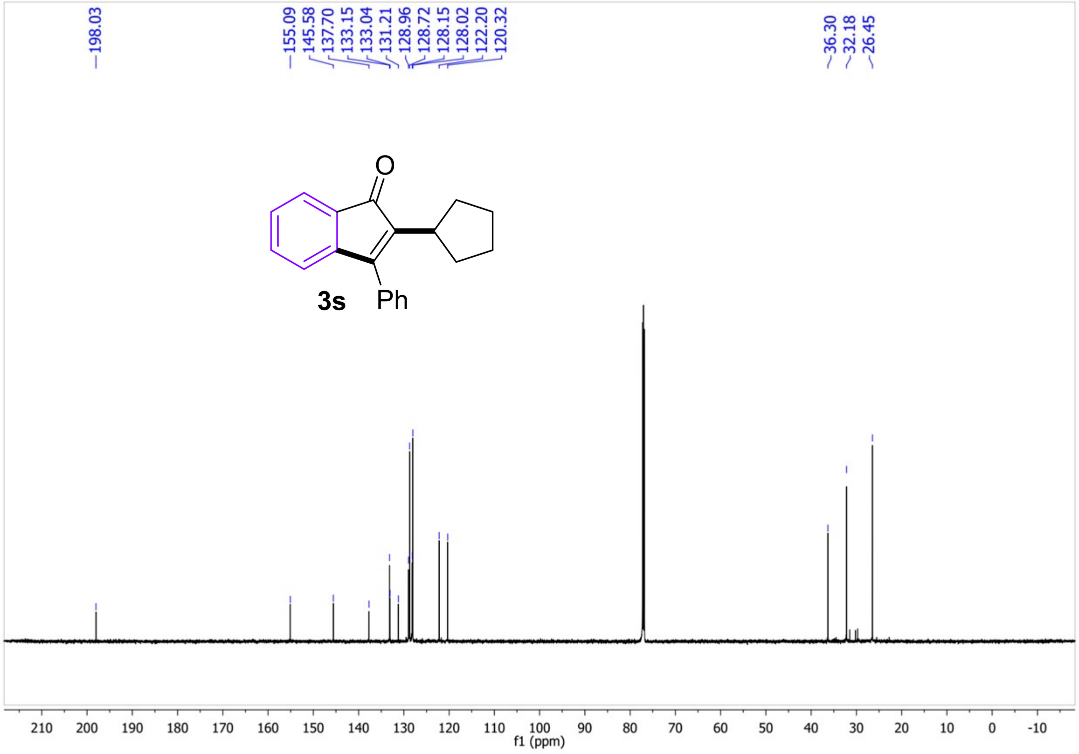

^{1}H NMR of **3s** in $CDCl_3$ (600 MHz, $CDCl_3$)

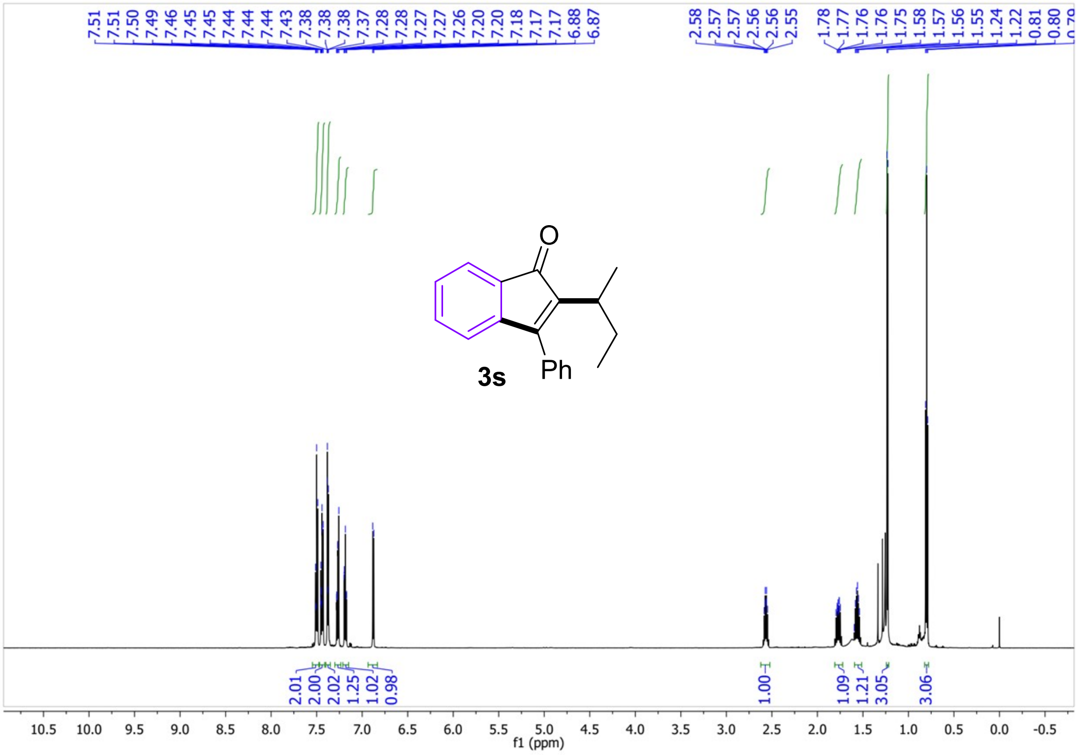

^{13}C NMR of **3s** in $CDCl_3$ (150 MHz, $CDCl_3$)

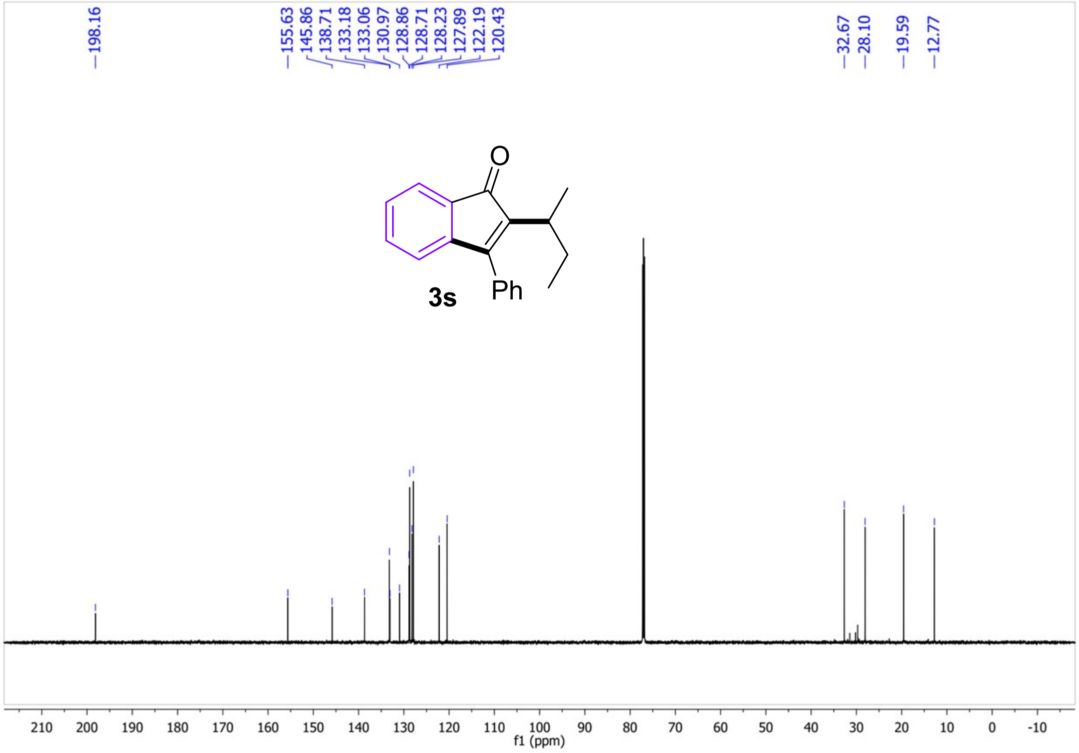

^{1}H NMR of **3t** in $CDCl_3$ (600 MHz, $CDCl_3$)

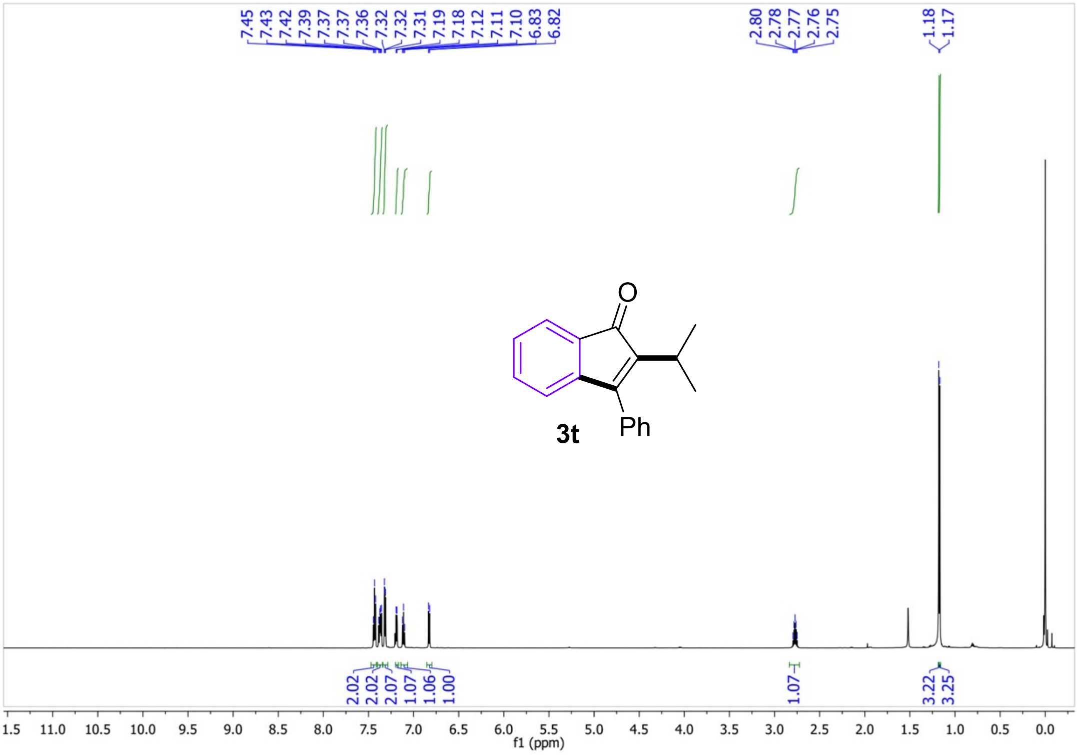

^{13}C NMR of **3s** in $CDCl_3$ (150 MHz, $CDCl_3$)

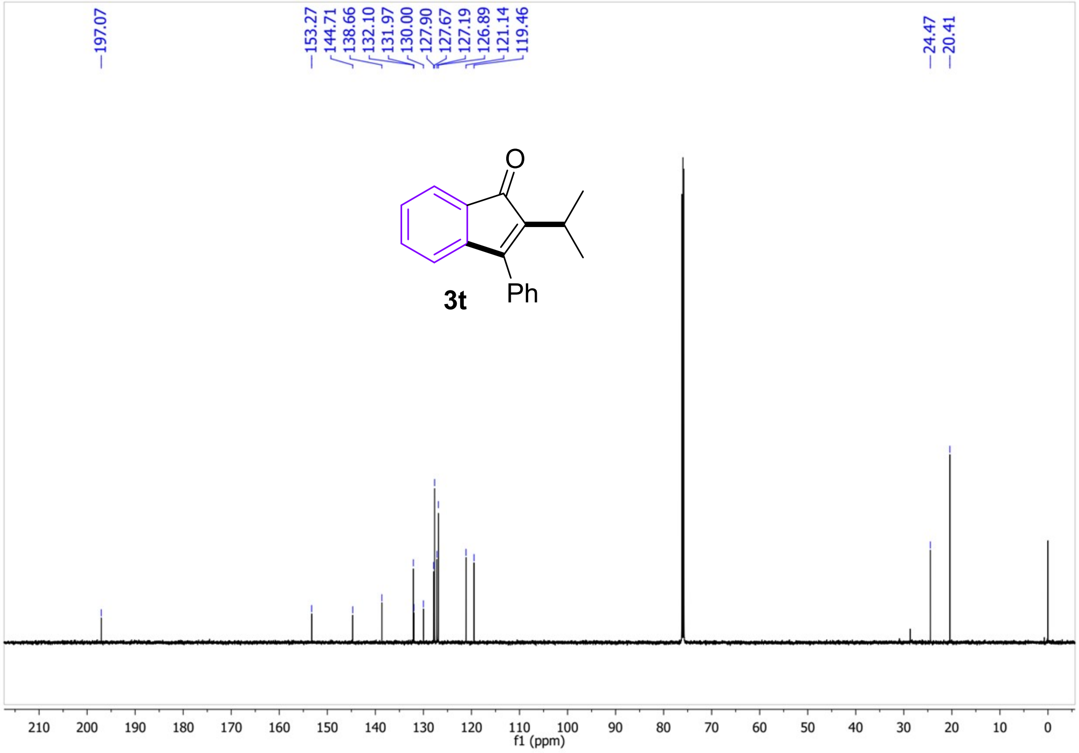

Supplement: RA-015-D5RA05436B-s001 [file RA-015-D5RA05436B-s001.pdf]
